# Transcriptomic analysis of reduced thermotolerance in *Arabidopsis* seedlings lacking the metacaspase AtMC1

**DOI:** 10.3389/fpls.2026.1873366

**Published:** 2026-09-09

**Authors:** Lin Chen, Xiaochun Ge

**Affiliations:** State Key Laboratory of Genetics and Development of Complex Phenotypes, School of Life Sciences, Fudan University, Shanghai, China

**Keywords:** *Arabidopsis thaliana*, AtMC1, heat stress, thermotolerance, transcriptome analysis

## Abstract

More frequent short-term episodes of extreme high temperatures caused by global warming seriously threaten plant growth and development around the world. Although heat stress can occur throughout the plant life cycle, exposure during the seedling stage severely impacts subsequent plant growth and productivity. In this study, we found that the *Arabidopsis* metacaspase mutant *atmc1* exhibited a heat-sensitive phenotype at the seedling stage, and restoration of *AtMC1* expression in the *atmc1* substantially rescued the heat-sensitive phenotype, indicating an important role of AtMC1 in thermotolerance. To investigate the underlying molecular responses associated with AtMC1 under heat stress, RNA sequencing was performed to compare the transcriptomic profiles of wild-type and *atmc1* seedlings under normal and heat treatment conditions. Gene set enrichment analysis (GSEA) and Kyoto Encyclopedia of Genes and Genomes (KEGG) pathway analyses identified key genes related to *heat acclimation*, *protein folding*, and endoplasmic reticulum-associated degradation (ERAD). Genes involved in reactive oxygen species (ROS) homeostasis also displayed differential expression patterns under heat stress. In addition, 45 transcription factors belonging to the ERF, HSF, WRKY, NAC, and MYB families were differentially expressed between wild-type and *atmc1* in response to heat stress. Protein-protein interaction analysis revealed 27 key heat-responsive genes, most of which were heat-induced but exhibited attenuated upregulation in *atmc1*. Collectively, our findings provide transcriptomic insights into the heat stress responses associated with loss of *AtMC1* in *Arabidopsis* seedlings and provide a foundation for future mechanistic studies of AtMC1-mediated thermotolerance.

## 1 Introduction

Global warming has increased the frequency and intensity of heat stress events, posing a major threat to plant growth, development, and crop productivity worldwide. Heat stress can cause protein denaturation and aggregation, reduce enzyme activity, disrupt organelle function, and promote the accumulation of toxic compounds such as ROS, ultimately leading to severe cellular damage or even plant death (Ding et al., 2020; Mittler et al., 2022; Kan et al., 2023). As sessile organisms, plants cannot escape unfavorable environmental conditions. Therefore, elucidating the molecular basis underlying plant responses to high temperatures is of considerable theoretical and practical importance.

Plant thermotolerance is generally classified into two distinct yet partially overlapping types: basal thermotolerance and acquired thermotolerance. Basal thermotolerance refers to the intrinsic ability of plants to survive direct heat shock without prior acclimation, whereas acquired thermotolerance describes the enhanced ability to withstand otherwise lethal heat shock following prior exposure to moderate heat stress, a process known as heat acclimation (Larkindale et al., 2005; Larkindale and Vierling, 2008; Bokszczanin et al., 2013).

The regulatory network governing systemic heat stress (HS) response in *Arabidopsis* has been progressively elucidated (Ding et al., 2020). Heat shock factors (HSFs) and heat shock proteins (HSPs) play central roles in the activation of heat shock responses (HSRs) (Ohama et al., 2017; Ding et al., 2020). Signal molecules including Ca^2+^, ROS, NO, and phytohormones, function upstream of HSFs to mediate heat signaling pathways (Volkov et al., 2006; Liu et al., 2007, Liu et al., 2008; Xuan et al., 2010; Finka et al., 2012; Guan et al., 2013; Huang et al., 2021; He et al., 2022; Luo et al., 2022; Saini et al., 2022). Additionally, unfolded protein responses (UPRs) in the endoplasmic reticulum (ER) (Wang et al., 2007; Sun et al., 2013; Ruberti et al., 2015; Ding et al., 2020) play a crucial role in HS responses (Kan et al., 2023). When UPR pathways fail to restore proper protein folding and ER homeostasis within a stipulated time frame, excessive misfolded proteins undergo ubiquitination and subsequent proteasomal degradation via the ERAD pathway (Bernales et al., 2006; Liu and Li, 2014; Strasser, 2018). Beyond protein quality control, plants must also counteract the excessive accumulation of ROS during heat stress. Key enzymes in the antioxidant defense system, including superoxide dismutase (SOD), catalase (CAT), ascorbate peroxidase (APX), glutathione peroxidase (GPX), glutathione reductase (GR), glutathione S-transferases (GSTs), monodehydroascorbate reductase (MDHAR), and dehydroascorbate reductase (DHAR), are essential for protecting cells and maintaining cellular hemostasis under stress conditions (Dietz, 2011; Kang et al., 2019; Dvorak et al., 2020; Foyer and Noctor, 2020; Mir and Khah, 2024). Notably, increased CAT and SOD activities have been shown to significantly improve plant thermotolerance (Wu et al., 2012; Qiao et al., 2015; Liao et al., 2023; Wang et al., 2023a, Wang et al., 2023b; Xie et al., 2024).

To date, the characterization of heat-sensitive mutants in *Arabidopsis has* largely converged on three interconnected pathways: transcriptional regulation, molecular chaperone systems, and ROS detoxification. Class A heat shock factors, particularly HSFA1s and HSFA2, act as master regulators of HSRs, and disruption of these genes markedly compromises basal and/or acquired thermotolerance (Prändl et al., 1998; Li et al., 2005; Charng et al., 2007; Kotak et al., 2007; Schramm et al., 2008; Ikeda et al., 2011; Liu et al., 2011; Kolmos et al., 2014; Lin et al., 2018; Yao et al., 2023). Likewise, the heat shock proteins HSP101 and HSP70, together with their co-chaperones, are essential for proteostasis under elevated temperatures, with corresponding mutants exhibiting pronounced heat hypersensitivity (Szabo et al., 1994; Glover and Lindquist, 1998; Morimoto, 1998; Hong and Vierling, 2000; Charng et al., 2006, Charng et al., 2007; Zhang et al., 2010; Jungkunz et al., 2011; Fu et al., 2015; Leng et al., 2017; Fernandez-Bautista et al., 2018; McLoughlin et al., 2019; Mondal et al., 2023). In addition, loss of the cytosolic ascorbate peroxidase APX2 impairs heat acclimation, underscoring the importance of ROS homeostasis in thermotolerance (Ogawa et al., 2007; Larkindale and Vierling, 2008).

Metacaspases (MCs) are cysteine proteases that share structural similarity with animal caspases but differ in substrate specificity and regulatory mechanisms (Minina et al., 2017; Huh, 2022). In plants, MCs have been implicated in diverse biological processes, including programmed cell death (Coll et al., 2010; Minina et al., 2017; Asqui et al., 2018), immune response (Wang et al., 2021; Pi et al., 2025; Salguero-Linares et al., 2025; Hu et al., 2026), and senescence (Coll et al., 2014; Ruiz-Solani et al., 2023). However, their roles in abiotic stress adaptation remain poorly understood, particularly in the context of heat stress. Among metacaspase family members, *Arabidopsis* metacaspase 1 (AtMC1) is recognized as a positive regulator of pathogen-triggered programmed cell death, functioning antagonistically with AtMC2 to fine-tune the hypersensitive response (Coll et al., 2010). Loss of AtMC1 disrupts NLR protein turnover and causes autoimmune phenotypes (Salguero-Linares et al., 2025). Beyond immunity, AtMC1 has also been shown to exert a pro-survival, homeostatic function in aging plants, acting in parallel with autophagy and contributing to stress granule dynamics and protein aggregate clearance (Coll et al., 2014; Ruiz-Solani et al., 2023). However, whether AtMC1 contributes to plant thermotolerance remains largely unknown.

In this study, we found that the *atmc1* mutant exhibited reduced thermotolerance compared with the wild type (WT). Estradiol-induced expression of *AtMC1* in the mutant restored heat tolerance, providing genetic evidence that AtMC1 contributes to thermotolerance. In addition, the *atmc1* mutant showed altered H_2_O_2_ accumulation under elevated temperatures. To investigate the molecular basis of this thermosensitive phenotype, we performed transcriptome profiling of *atmc1* and WT seedlings under both control and heat stress conditions. Comparative RNA-seq analysis revealed that loss of *AtMC1* led to extensive transcriptional reprogramming of heat-responsive genes, particularly those involved in proteostasis and ROS metabolism. Furthermore, we identified hub genes and co-expression modules associated with the heat response in the *atmc1* mutant, providing promising candidates for future mechanistic studies of AtMC1-mediated thermotolerance.

## 2 Materials and methods

### 2.1 Plant materials and heat tolerance evaluation

*Arabidopsis* seeds used in this study are of the Columbia-0 (Col-0) ecotype. The T-DNA insertion line *atmc1* (*At1g02170*, CS68732) was obtained from the Arabidopsis Biological Resource Center (ABRC) and was originally donated by Jeff Dangl (Coll et al., 2010). All plants were cultivated in a growth chamber at 22 °C under a 16 h light/8 h dark photoperiod.

**Figure 1:**
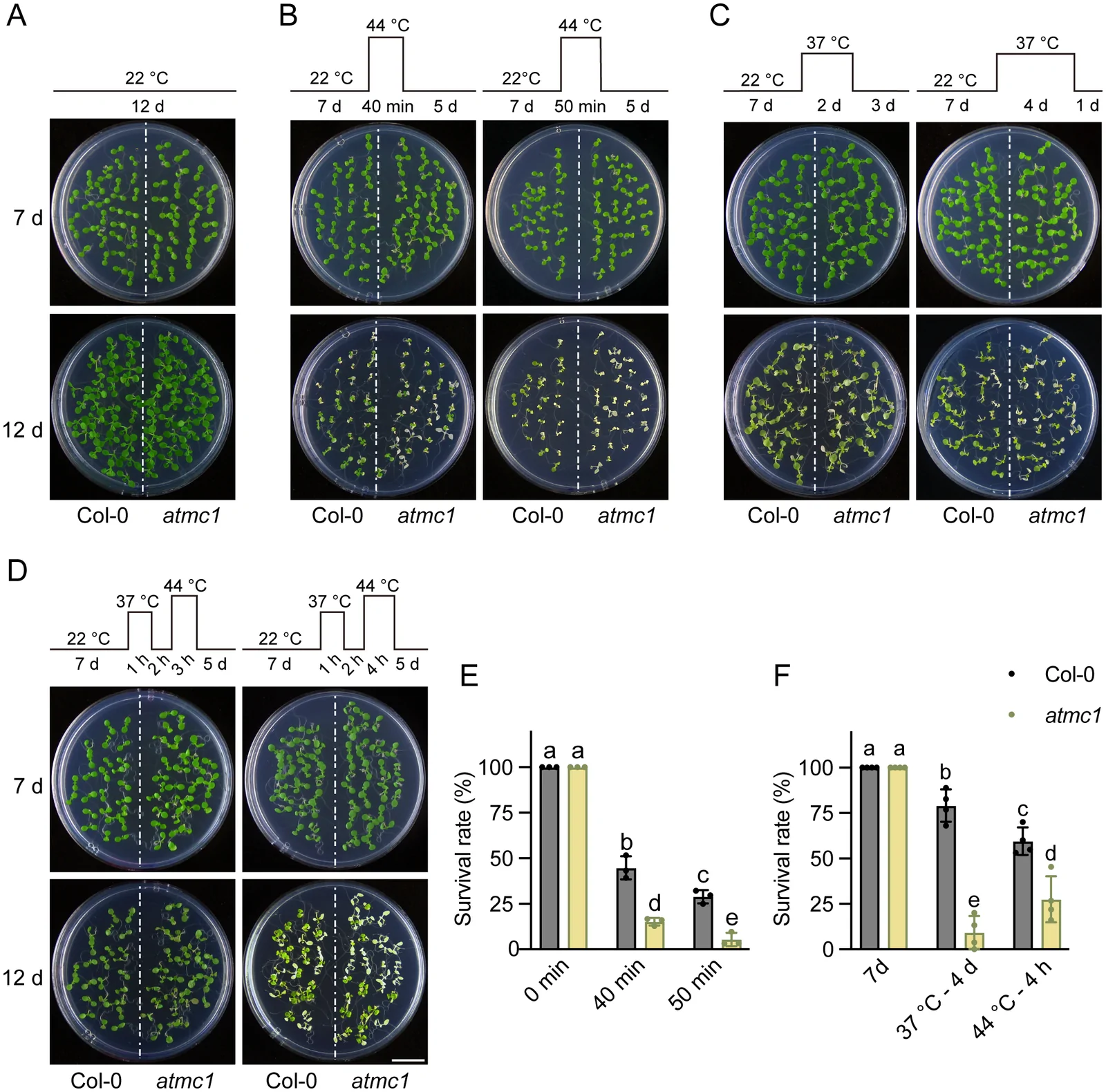
The *atmc1* mutant exhibits reduced thermotolerance at the seedling stage. Phenotypes of Col-0 and *atmc1* seedlings under **(A)** normal conditions, **(B)** basal thermotolerance assay with acute heat shock, **(C)** prolonged moderate heat stress, and **(D)** acquired thermotolerance assay. **(E)** Quantification of survival rates of seedlings shown in **(A, B)**. **(F)** Quantification of survival rates corresponding to **(C, D)**. The upper panels show schematic diagrams of the experimental procedures. Scale bar = 1 cm. Data are shown as mean ± SD (*n* ≥ 3 biological replicates, with approximately 30 seedlings per replicate). Different letters indicate statistically significant differences determined by two-way ANOVA followed by Tukey’s multiple comparison test (*P* < 0.05).

**Figure 2:**
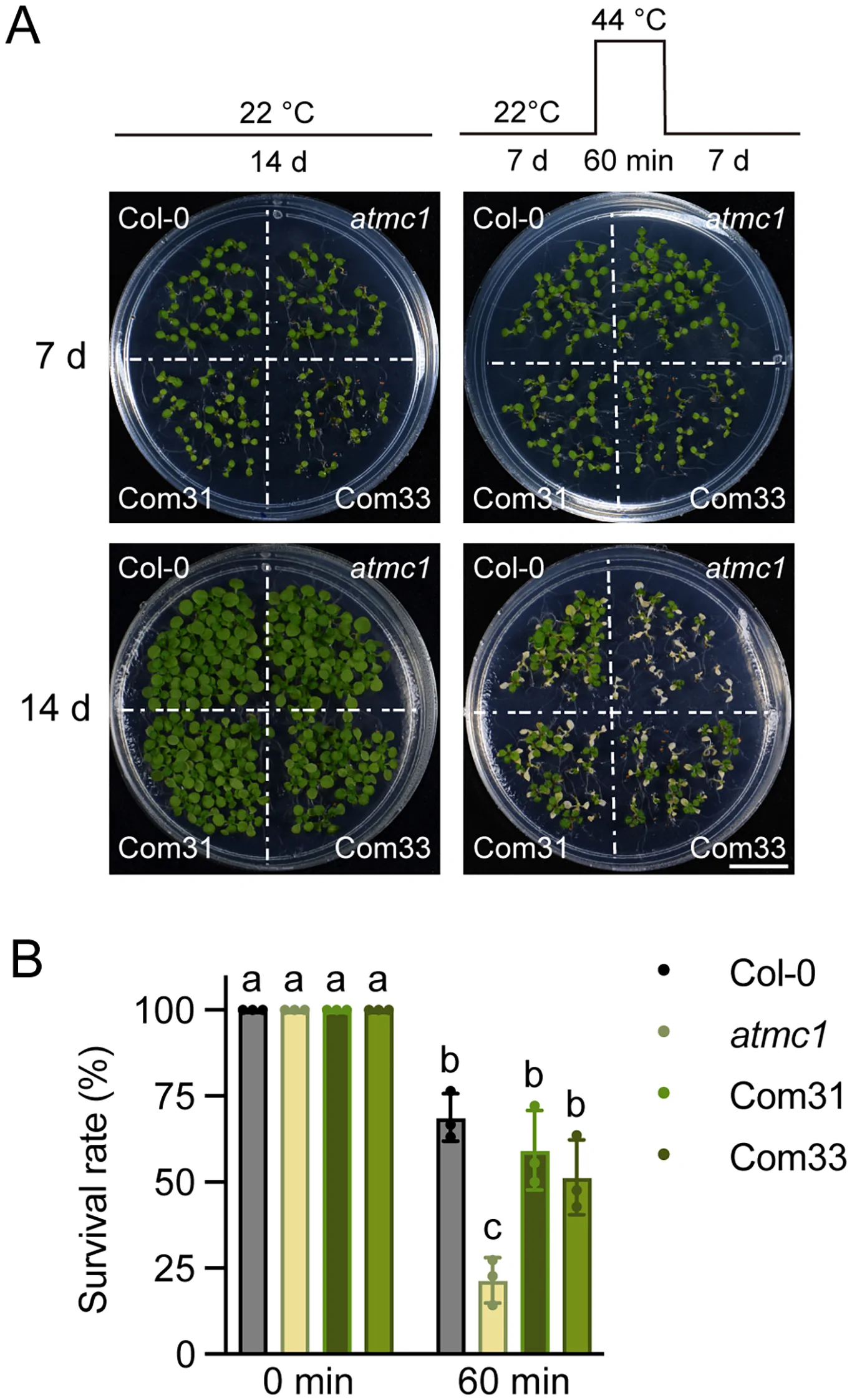
Estradiol-induced *AtMC1* expression rescues the heat-sensitive phenotype of *atmc1*. **(A)** Seedling phenotypes of Col-0, *atmc1*, Com31 and Com33 under control conditions and after acute heat shock in the basal thermotolerance assay. The upper panels show schematic diagrams of the procedures. Scale bar = 1 cm. **(B)** Quantification of survival rates corresponding to **(A)**. Only normally germinated 5-day-old seedlings were used for analysis. Data are shown as mean ± SD (*n* ≥ 3 biological replicates, with approximately 20 seedlings per replicate). Different letters indicate statistically significant differences determined by two-way ANOVA followed by Tukey’s multiple comparison test (*P* < 0.05).

For heat tolerance assays, 7-day-old seedlings grown on 1/2 MS medium were subjected to heat stress according to the experimental procedures shown in **Figures 1** and **2**. For complementation experiment, 5-day-old pER8::*AtMC1*-HA/*atmc1* seedlings were uniformly treated with 1 mL of 10 μM estradiol per 6-cm Petri dish. The solution was allowed to absorb into the medium within 2 days before heat treatment.

### 2.2 Construction of transformation vectors and generation of complementation lines

To generate the estradiol-inducible pER8::*AtMC1*-HA construct, the *AtMC1* coding sequence and 3×HA tag were amplified using the primer pairs MC1(XhoI) F1/MC1 R1 and 3×HA-F2/3×HA(SpeI) R2, respectively, as listed in **Supplementary Table 1**. The resulting PCR products were assembled into the plant binary vector pER8 using the NovoRec^®^ Plus One-Step PCR Cloning Kit (Cat. No. NR005; Novoprotein Scientific Inc., Shanghai, China), according to the manufacturer’s instructions. The recombinant plasmid was introduced into *Agrobacterium tumefaciens* strain GV3101. The resulting pER8::*AtMC1*-HA construct was introduced into the *atmc1* mutant by *Agrobacterium*-mediated floral dip transformation as previously described (Clough and Bent, 1998). Transgenic plants were screened on 1/2 MS medium supplemented with 80 μg/mL hygromycin. The homozygous T2 transgenic lines were used for complementation analysis.

### 2.3 DAB staining and quantification of H_2_O_2_ content

5-day-old *Arabidopsis* seedlings grown on 1/2 MS medium at 22 °C were subjected to heat treatment at 37 °C or 44 °C for 1 h. Control and heat-treated seedlings were then incubated in freshly prepared DAB staining solution containing 1 mg/mL 3,3’-diaminobenzidine (DAB), 0.05% (v/v) Tween 20, and 10 mM Na_2_HPO_4_ at room temperature in the dark for 6–8 h, followed by decolorization in an ethanol: acetic acid: glycerol solution (3:1:1, v/v/v), as previously described (Daudi and O'Brien, 2012). DAB-stained seedlings were imaged using a stereomicroscope (MSD202-B8, Guangdong, China). Staining and imaging conditions were kept consistent across all samples, including exposure time and microscope settings.

The endogenous H_2_O_2_ content was determined using the titanium sulfate colorimetric method (Satterfield and Bonnell, 1955) with slight modifications. Briefly, approximately 0.1 g of seedlings were homogenized in 1 mL ice-cold acetone. After centrifugation, the supernatant was reacted with 1/10 volume of 5% titanium sulfate and 1/5 volume of concentrated ammonia solution to form a titanium-peroxide complex. The resulting precipitate was collected by centrifugation, repeatedly washed with acetone to remove pigments, and dissolved in 2 mM H_2_SO_4_. The absorbance of the solution was measured at 415 nm. H_2_O_2_ content was calculated from the standard curve (**Supplementary Figure 1**) and normalized to the fresh weight of plant tissues.

### 2.4 Sample collection

For RNA-seq analysis, Col-0 and *atmc1* seedlings were grown side by side on 1/2 MS medium at 22 °C for 7 days and then subjected to heat stress at 37 °C for 0 and 1 h. To examine the expression pattern of *AtMC1* in response to heat stress, 7-day-old Col-0 seedlings were subjected to heat stress at 37 °C for 0, 0.25, 0.5, 1, and 3 h. Approximately 200 mg of seedlings were harvested at each time point, immediately snap-frozen in liquid nitrogen, and stored at −80 °C until further experiments.

### 2.5 RNA extraction and RT-qPCR

Total RNA was extracted using TRIzol reagent (Takara, Japan) according to the manufacturer’s instructions. RNA concentration was measured using a NanoDrop 2000 spectrophotometer (Thermo Fisher Scientific, USA). First-strand cDNA was synthesized with the PrimeScript™ RT Reagent Kit with gDNA Eraser (Perfect Real Time) (Takara, Japan), according to the manufacturer’s instructions. RT-qPCR was performed using a CFX96™ Real-Time PCR Detection System (Bio-Rad, USA) with TB Green^®^ Premix Ex Taq™ II (Takara, Japan). Relative expression levels were calculated using the 2^-ΔΔCt^ method. The primers used in this study are listed in **Supplementary Table 1**. The amplification protocol consisted of an initial denaturation at 95 °C for 30 s, followed by 40 cycles of 95 °C for 5 s and 60 °C for 34 s. For RT-qPCR validation, three biological replicates were analyzed, including three technical replicates for each biological replicate. Other RT-qPCR experiments included technical replicates only, unless otherwise indicated. *ACTIN2* was used as the internal reference gene for normalization, as its expression has been reported to remain relatively stable under heat stress in *Arabidopsis* (Li et al., 2019).

### 2.6 RNA sequencing and data analysis

RNA integrity, concentration, and purity were assessed before library preparation. A total of 12 cDNA libraries were constructed using the VAHTS Universal V6 RNA-seq Library Prep Kit and sequenced on the Illumina NovaSeq™ 6000 platform by OE Biotech Co., Ltd. (Shanghai, China) using a paired-end 150-bp (PE150) sequencing strategy.

Raw sequencing reads were assessed using FastQC (v0.11.9) and processed using fastp (v0.20.1) to remove adapter sequences, low-quality reads, and reads containing excessive ambiguous bases. Sequencing saturation, library quality, and genome coverage were assessed using RSeQC (v4.0.0). The resulting clean reads were aligned to the *Arabidopsis thaliana* reference genome (TAIR10.1) using HISAT2 (v2.1.0) (Kim et al., 2015). Alignment files were sorted using SAMtools (v1.9), and gene-level read counts were generated using HTSeq-count (v0.11.2) (Anders et al., 2015). Genes with zero counts across all samples were excluded from downstream analyses. Pearson’s correlation coefficient (PCC), principal component analysis (PCA), and hierarchical clustering analysis (HCA) were performed to assess sample relationships and reproducibility. Differential expression analysis was performed using DESeq2 (v1.22.2) based on raw read counts. Genes with |log_2_FC| > 1 and *q* < 0.05 were considered differentially expressed genes (DEGs), where FC denotes fold change. FPKM values were calculated for visualization and comparative expression analysis. Functional enrichment analyses, including Gene Ontology (GO), KEGG pathway, and GSEA were performed using the OECloud online platform (https://cloud.oebiotech.com). Transcription factors (TFs) and their target genes were identified from the DEG dataset, and their relationships were visualized using Sankey diagrams in OECloud. Protein–protein interaction (PPI) networks were constructed using the STRING database and visualized with Cytoscape (v3.9.1).

### 2.7 Statistical analysis

Statistical analyses were performed using GraphPad Prism (version 11.0). For experiments involving a single variable, statistical significance was assessed using one-way analysis of variance (ANOVA) followed by Tukey’s multiple-comparisons test unless otherwise indicated. Experiments involving two independent variables (genotype and treatment) were analyzed using two-way ANOVA followed by Tukey’s multiple-comparisons test. Data are presented as mean ± standard deviation (SD).

## 3 Results

### 3.1 The *atmc1* mutant exhibited compromised basal and acquired thermotolerance

Since metacaspases have been well documented to mediate programmed cell death in response to biotic stresses (Coll et al., 2010; Coll et al., 2014; Wang et al., 2021; Ruiz-Solani et al., 2023; Salguero-Linares et al., 2025), we hypothesized that they might also participate in abiotic stress responses. As AtMC1 is a key member of the metacaspase family, we investigated its potential involvement in heat stress responses by evaluating basal and acquired thermotolerance of the *atmc1* mutant.

Under normal growth conditions, no visible morphological differences were observed between 7-day-old *atmc1* and Col-0 seedlings (**Figure 1A**). Upon acute heat shock at 44 °C for 40 or 50 min followed by 5 days of recovery, the *atmc1* mutant exhibited aggravated heat sensitivity, accompanied by more severe chlorosis and significantly lower survival relative to Col-0 (**Figures 1B, E**). Similarly, exposure to prolonged moderate heat stress at 37 °C for 2 or 4 days led to more severe leaf curling and wilting, as well as reduced survival in *atmc1* seedlings (**Figures 1C, F**). We next examined acquired thermotolerance using heat acclimation regimes. Despite pre-acclimation, the *atmc1* mutant remained more heat-sensitive than Col-0, yet the difference was less pronounced than in the basal thermotolerance assay (**Figures 1D, F**).

### 3.2 Inducible *AtMC1* complementation rescues heat sensitivity of the *atmc1* mutant

To further validate the role of *AtMC1* in thermotolerance, we generated estradiol-inducible complementation lines. Two independent pER8::*AtMC1*-HA/*atmc1* lines (Com31 and Com33) were used for subsequent thermotolerance analysis. 5-day-old seedlings were treated with 10 μM estradiol for 2 days to induce *AtMC1* expression before heat treatment. Without heat treatment, induced *AtMC1* expression had no effect on seedling survival (**Figure 2A**). After acute heat shock at 44 °C followed by 7 days of recovery, the *atmc1* mutant exhibited reduced survival, whereas both complementation lines showed survival rates comparable to Col-0 and significantly higher than those of the mutant (**Figures 2A, B**). These results indicate that inducible restoration of *AtMC1* expression rescues the heat-sensitive phenotype of the *atmc1* mutant and provides additional genetic evidence for the role of *AtMC1* in thermotolerance.

### 3.3 Transcriptome sequencing and quality assessment

To investigate the molecular basis of the heat-sensitive phenotype of the *atmc1* mutant, we performed RNA-seq on Col-0 and *atmc1* seedlings under control (CK) and heat treatment (HT) conditions. Considering that transcriptional reprogramming occurs rapidly following stress perception, seedlings were collected 1 h after heat treatment at 37 °C. Three biological replicates were included for each group, generating 12 cDNA libraries sequenced on the Illumina NovaSeq™ 6000 platform. A total of 583.55 million raw reads (86.29 Gb) were obtained, with 562.65 million clean reads (83.20 Gb) retained after filtering. Each sample yielded 6.59–7.07 Gb of clean data, with Q30 values ranging from 93.86 to 94.75%, and genome mapping rates ranging from 98.83 to 98.92% (**Supplementary Table 2**). Genes with zero counts across all samples were removed, and a statistical summary of expressed genes is provided in **Supplementary Table 3** and **Supplementary Figure 2A**. Expression distributions were consistent across samples (**Supplementary Figures 2B, C**). PCC, PCA, and HCA further demonstrated high reproducibility among biological replicates (*r* > 0.99) and clear separation among treatment and genotype groups (**Supplementary Figures 2D–F**). These results confirmed the high quality and reproducibility of the RNA-seq data for subsequent analyses.

### 3.4 Identification of DEGs in response to heat stress

**Figure 3:**
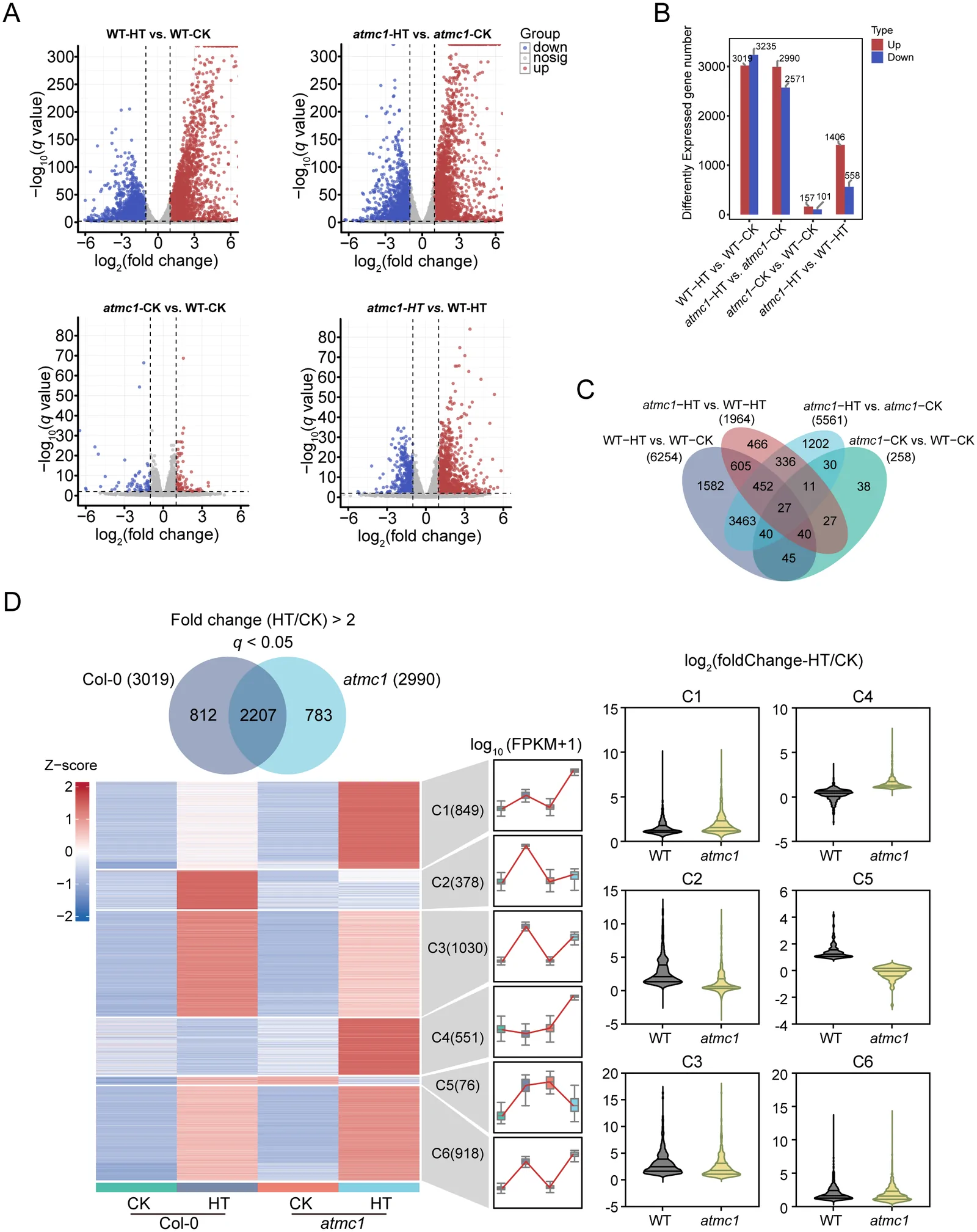
Global identification of DEGs. **(A)** Volcano plots showing DEGs identified in four comparisons. **(B)** Numbers of upregulated and downregulated DEGs in each comparison. **(C)** Venn diagram showing shared and unique DEGs across comparisons. **(D)** Venn diagram and heatmap showing 3,802 heat-induced DEGs identified in Col-0 and/or *atmc1*. Heat-induced DEGs were divided into six expression clusters (C1–C6). Boxplots display expression levels, and violin plots show the distribution of log_2_(fold change-HT/CK) for genes in each cluster.

DEGs were defined using thresholds of |log_2_FC| > 1 and *q* < 0.05. Four pairwise comparisons identified 6,254 (3,019 upregulated and 3,235 downregulated), 5,561 (2,990 upregulated and 2,571 downregulated), 258 (157 upregulated and 101 downregulated), and 1,964 (1,406 upregulated and 558 downregulated) DEGs in WT-HT vs. WT-CK, *atmc1*-HT vs. *atmc1*-CK, *atmc1*-CK vs. WT-CK, and *atmc1*-HT vs. WT-HT (**Figures 3A, B**; **Supplementary Data Sheet 1**; **Supplementary Table 4**). Only 27 DEGs overlapped across all four comparisons. In addition, 1,582, 1,202, and 466 DEGs were unique to the WT-HT vs. WT-CK, *atmc1*-HT vs. *atmc1*-CK, and *atmc1*-HT vs. WT-HT comparisons, respectively, while 452 DEGs were shared among three comparisons (**Figure 3C**; see also **Supplementary Data Sheet 1**).

To further characterize transcriptional responses to heat stress, we compared the heat-responsive genes between Col-0 and *atmc1*. Heat treatment significantly upregulated 3,019 genes in Col-0 and 2,990 genes in *atmc1* (HT/CK fold change > 2, *q* < 0.05). Of these, 2,207 genes were commonly induced in both genotypes, whereas 812 and 783 genes were uniquely induced in Col-0 and *atmc1*, respectively (**Figure 3D**; **Supplementary Data Sheet 1**; **Supplementary Table 5**). These heat-responsive genes were grouped into six clusters (C1–C6) based on their expression patterns. Violin plots of log_2_-transformed HT/CK fold changes revealed distinct heat-responsive patterns between the two genotypes. Genes in C1 and C6 were more strongly induced in *atmc1*, whereas those in C2 and C3 showed attenuated induction in *atmc1*. This impaired transcriptional response may contribute to the compromised heat tolerance of the *atmc1* mutant. Interestingly, genes in C4 were preferentially induced in *atmc1* with little response in Col-0. In contrast, genes in C5 were induced specifically in Col-0 but repressed in *atmc1*. Overall, these results indicate that loss of AtMC1 is associated with distinct patterns of heat-responsive gene expression.

### 3.5 Functional enrichment analysis of DEGs

To characterize the transcriptional responses to heat stress, GO and KEGG enrichment analyses were performed for DEGs from the four pairwise comparisons (**Supplementary Data Sheet 2**).

#### 3.5.1 GO enrichment

**Figure 4:**
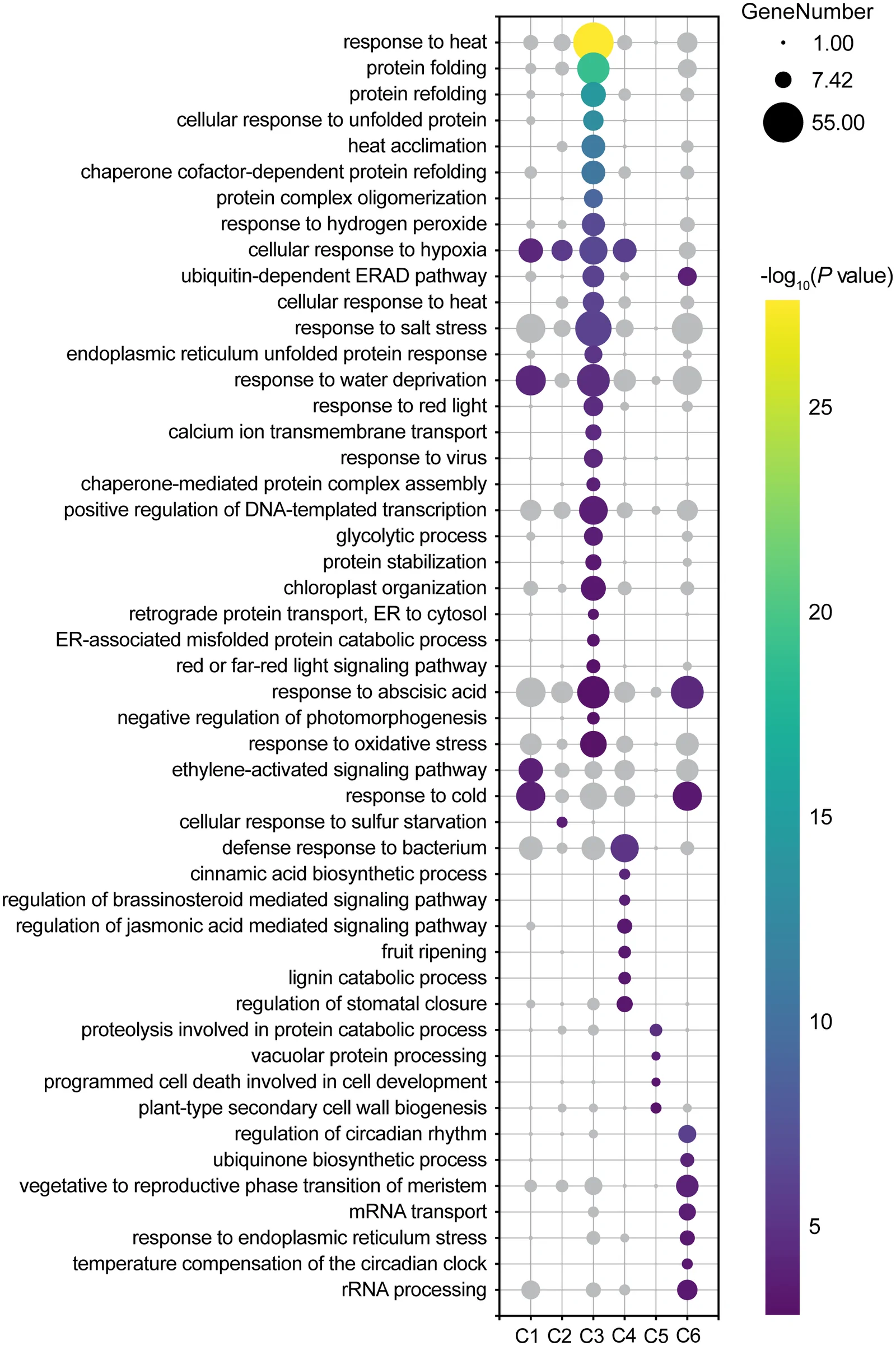
Bubble plot of GO enrichment analysis for six clusters (C1–C6). Bubble size indicates gene number. Colored bubbles show -log_10_(*P* value) for terms with *q* < 0.05, whereas gray bubbles represent non-significant terms.

Significantly enriched GO terms were identified using PopHits ≥ 5 and *q* < 0.05 (**Supplementary Table 6**), and representative GO terms are shown in **Supplementary Figure 3** and **Figure 4**. In WT-HT vs. WT-CK, DEGs were predominantly enriched in abiotic and biotic stress responses, including *response to heat* (GO:0009408), *response to water deprivation* (GO:0009414), *response to osmotic stress* (GO:0006970), *response to abscisic acid* (GO:0009737), and *defense response to fungu*s (GO:0050832). Upregulated genes were particularly enriched in *heat acclimation* (GO:0010286), *protein folding* (GO:0006457), *protein refolding* (GO:0042026), and oxidative stress-related processes including *response to oxidative stress* (GO:0006979) and *cellular oxidant detoxification* (GO:0098869), whereas downregulated genes were associated mainly with *RNA modification* (GO:0009451), hormone responses including *response to salicylic acid* (GO:0009751) and *response to auxin* (GO:0009733), and light responses such as *response to red light* (GO:0010114) (**Supplementary Figure 3A**). The *atmc1*-HT vs. *atmc1*-CK comparison showed similar enrichment of heat-, osmotic-, and ABA-responsive processes among upregulated genes, whereas downregulated genes were additionally enriched in developmental processes, including *plant epidermis development* (GO:0090558) and *glucosinolate biosynthetic process* (GO:0019761) (**Supplementary Figure 3B**). Under basal conditions, DEGs in *atmc1*-CK vs. WT-CK were mainly enriched in defense- and immunity-related processes, including *defense response to fungus*, *defense response to bacterium* (GO:0042742), *response to salicylic acid*, and *systemic acquired resistance* (GO:0009627). Defense-related secondary metabolic processes, including *indole glucosinolate biosynthetic process* (GO:0009759) and *camalexin biosynthetic process* (GO:0010120), were also enriched, while *response to heat* was among the downregulated genes (**Supplementary Figure 3C**). Following heat stress, DEGs in *atmc1*-HT vs. WT-HT showed distinct enrichment patterns. Upregulated genes were predominantly associated with defense and immune responses, including *defense response to bacterium*, *defense response to fungus*, and *systemic acquired resistance*, as well as hormone-related processes, including *response to salicylic acid*, *salicylic acid–mediated signaling pathway* (GO:0009862), *response to jasmonic acid* (GO:0009753), and *response to abscisic acid*. In contrast, downregulated genes were enriched in heat-responsive processes, including *response to heat*, *cellular response to heat* (GO:0034605), *heat acclimation*, *protein folding*, and *chaperone-mediated protein complex assembly* (GO:0051131). Developmental and light-related processes, including *tissue development* (GO:0009888), *root development* (GO:0048364), *response to red light*, and *response to light intensity* (GO:0009642), were also enriched among downregulated genes (**Supplementary Figure 3D**).

We further examined the functional enrichment of the six expression clusters (**Figure 4**; **Supplementary Table 7**). Clusters with weaker heat induction in *atmc1* were enriched in heat stress response and protein homeostasis processes, including *response to heat*, *heat acclimation*, *response to hydrogen peroxide*, and *protein folding*, as well as protein degradation processes such as the *ubiquitin-dependent ERAD pathway*, *protein catabolic process*, and *vacuolar protein processing*. Hormone-related processes, particularly *response to abscisic acid*, were also enriched. In contrast, C1 and C4, which showed stronger or preferential induction in *atmc1*, were enriched in hormone-related processes, including *ethylene-activated signaling pathway*, *regulation of brassinosteroid mediated signaling pathway*, and *regulation of stomatal closure*. Overall, these patterns were consistent with weaker induction of core heat-responsive processes and altered stress- and hormone-related responses in *atmc1* under heat stress.

#### 3.5.2 KEGG enrichment

**Figure 5:**
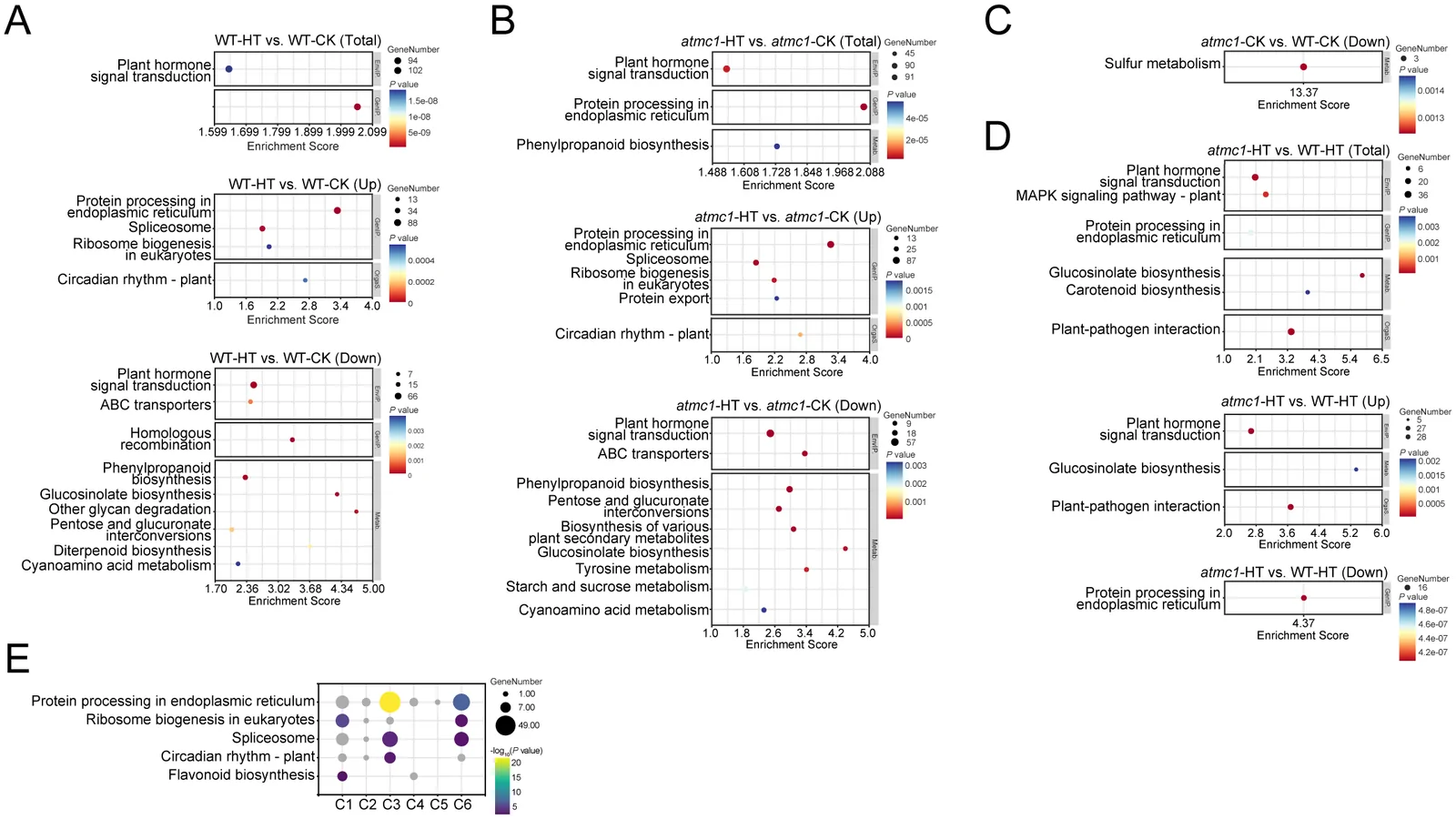
KEGG pathway enrichment analysis of DEGs. **(A)** WT-HT vs. WT-CK, **(B)** *atmc1*-HT vs. *atmc1*-CK, **(C)** *atmc1*-CK vs. WT-CK, **(D)** *atmc1*-HT vs. WT-HT, and **(E)** gene clusters. Bubble size indicates gene number. Colored bubbles show *P* value or -log_10_(*P* value) for pathways with *q* < 0.05, whereas gray bubbles represent non-significant pathways.

KEGG pathway enrichment analysis was performed for DEGs from all pairwise comparisons, with *q* < 0.05 considered significant (**Supplementary Table 8**; **Figure 5**). In WT-HT vs. WT-CK, DEGs were significantly enriched in *protein processing in endoplasmic reticulum* (ath04141) and *plant hormone signal transduction* (ath04075). Upregulated genes were predominantly enriched in pathways related to protein homeostasis and transcriptional regulation, including *protein processing in endoplasmic reticulum*, *spliceosome* (ath03040), *ribosome biogenesis in eukaryotes* (ath03008), and *circadian rhythm - plant* (ath04712). In contrast, downregulated genes were significantly enriched in pathways such as *plant hormone signal transduction*, *homologous recombination* (ath03440), *phenylpropanoid biosynthesis* (ath00940), *glucosinolate biosynthesis* (ath00966), and *other glycan degradation* (ath00511). The *atmc1*-HT vs. *atmc1*-CK comparison showed a broadly similar enrichment pattern, with additional enrichment of *phenylpropanoid biosynthesis* among all DEGs and *protein export* (ath03060) among upregulated genes. Downregulated genes were enriched in broader metabolic pathways, including *biosynthesis of various plant secondary metabolites* (ath00999) and *tyrosine metabolism* (ath00350). Under basal conditions, no significant KEGG pathways were identified among either the total or upregulated DEGs in atmc1-CK vs. WT-CK, whereas *sulfur metabolism* (ath00920) was enriched among the downregulated genes. Following heat stress, DEGs in *atmc1*-HT vs. WT-HT were enriched in *protein processing in the endoplasmic reticulum*, *MAPK signaling pathway–plant* (ath04016), and *plant–pathogen interaction* (ath04626). Downregulated genes were enriched in the heat-induced pathway *protein processing in endoplasmic reticulum*, whereas upregulated genes were enriched in pathways typically repressed by heat stress, including *plant hormone signal transduction* and *glucosinolate biosynthesis*.

We further examined KEGG pathway enrichment across the six gene clusters (**Figure 5E**; **Supplementary Table 9**). C1 was specifically enriched in *flavonoid biosynthesis*. Both C3 and C6 were enriched in *protein processing in endoplasmic reticulum* and *spliceosome* pathways. C3 was additionally enriched in *circadian rhythm*, whereas C6 was enriched in *ribosome biogenesis in eukaryotes*. No significant KEGG pathway enrichment was detected for C2, C4, or C5.

### 3.6 GSEA analysis

**Figure 6:**
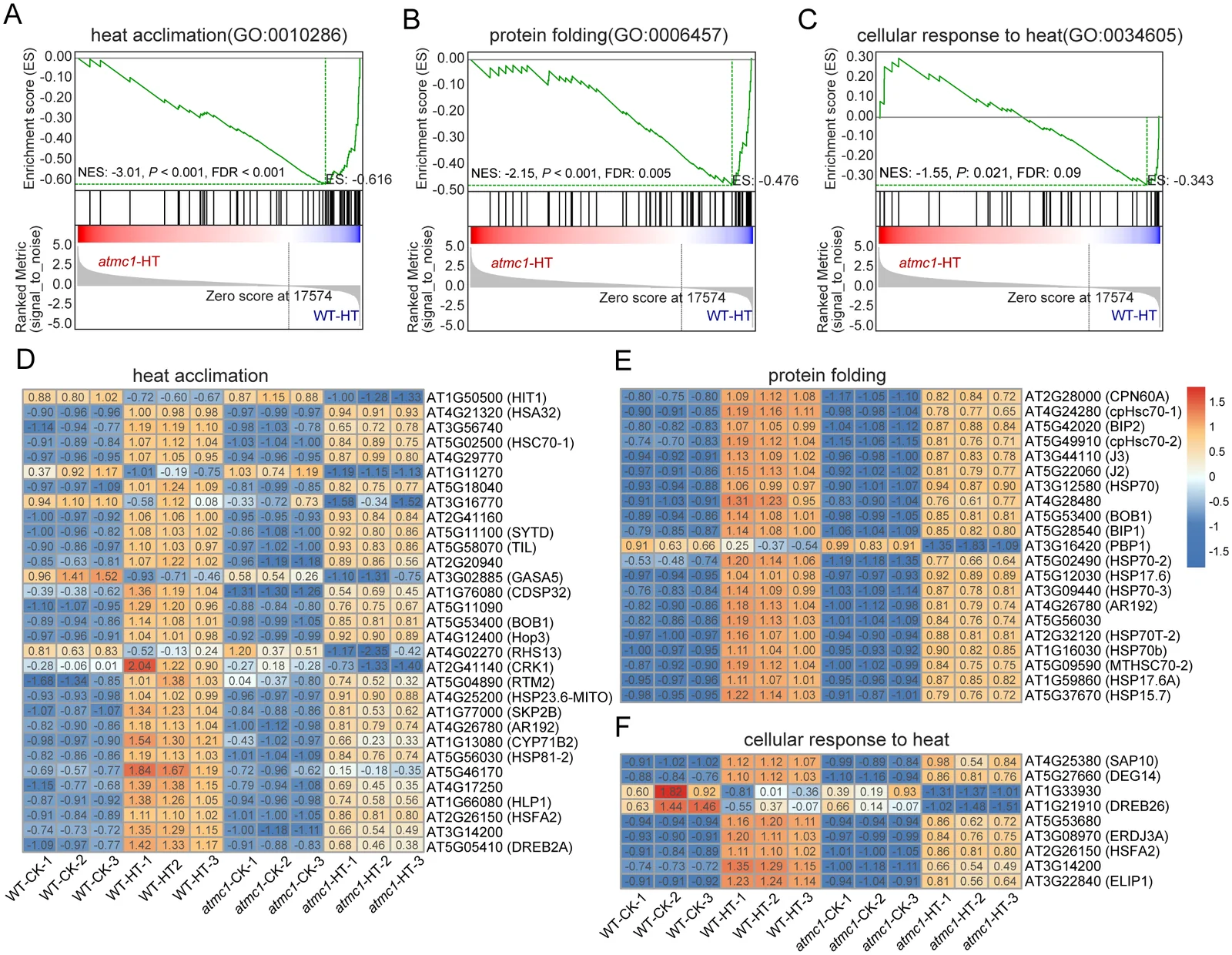
GSEA of BPs and heatmaps of related genes. GSEA was performed for **(A)** heat acclimation, **(B)** protein folding, and **(C)** cellular response to heat. Expression patterns of non-redundant core DEGs associated with each process are shown in **(D–F)**, respectively. The color scale represents the z-score normalized FPKM values.

GSEA enables the detection of coordinated but relatively modest expression changes across predefined gene sets. We therefore performed GSEA based on GO and KEGG annotations. Significant enrichment was defined as |NES| > 1, nominal *P* < 0.05, and *q* < 0.25. The results indicated a significant downregulation of gene sets associated with *heat acclimation*, *protein folding*, and *cellular response to heat*, and *protein processing in endoplasmic reticulum* in *atmc1*-HT vs. WT-HT. Notably, most core genes contributing to these enrichments were heat-induced genes whose expression increased in response to heat treatment in both genotypes but showed a reduced magnitude of induction in *atmc1* compared with WT (**Figure 6**; **Supplementary Figure 4**; **Supplementary Table 10**). Further examination of individual gene sets revealed distinct transcriptional changes in *atmc1*. Of the 33 core genes associated with *heat acclimation*, 31 were retained after excluding *AT4G29760* and *AT2G16575* due to low expression (FPKM < 1). *AT3G02885*/*GASA5* was downregulated, whereas *AT4G02270*/*RHS13* was specifically repressed in *atmc1* (**Figures 6A, D**). Of the 21 core genes associated with *protein folding*, all were heat-induced except *AT3G16420*/*PBP1*, which showed no significant response in Col-0 but was downregulated in *atmc1* (**Figures 6B, E**). Among the nine core genes associated with *cellular response to heat*, *AT1G21910*/*DREB26* was downregulated, whereas *AT1G33930* was specifically repressed in *atmc1* (**Figures 6C, F**). All 34 core DEGs enriched in protein processing in endoplasmic reticulum were heat-induced genes (**Supplementary Figure 4**).

### 3.7 The pathway of protein processing in endoplasmic reticulum under heat stress

**Figure 7:**
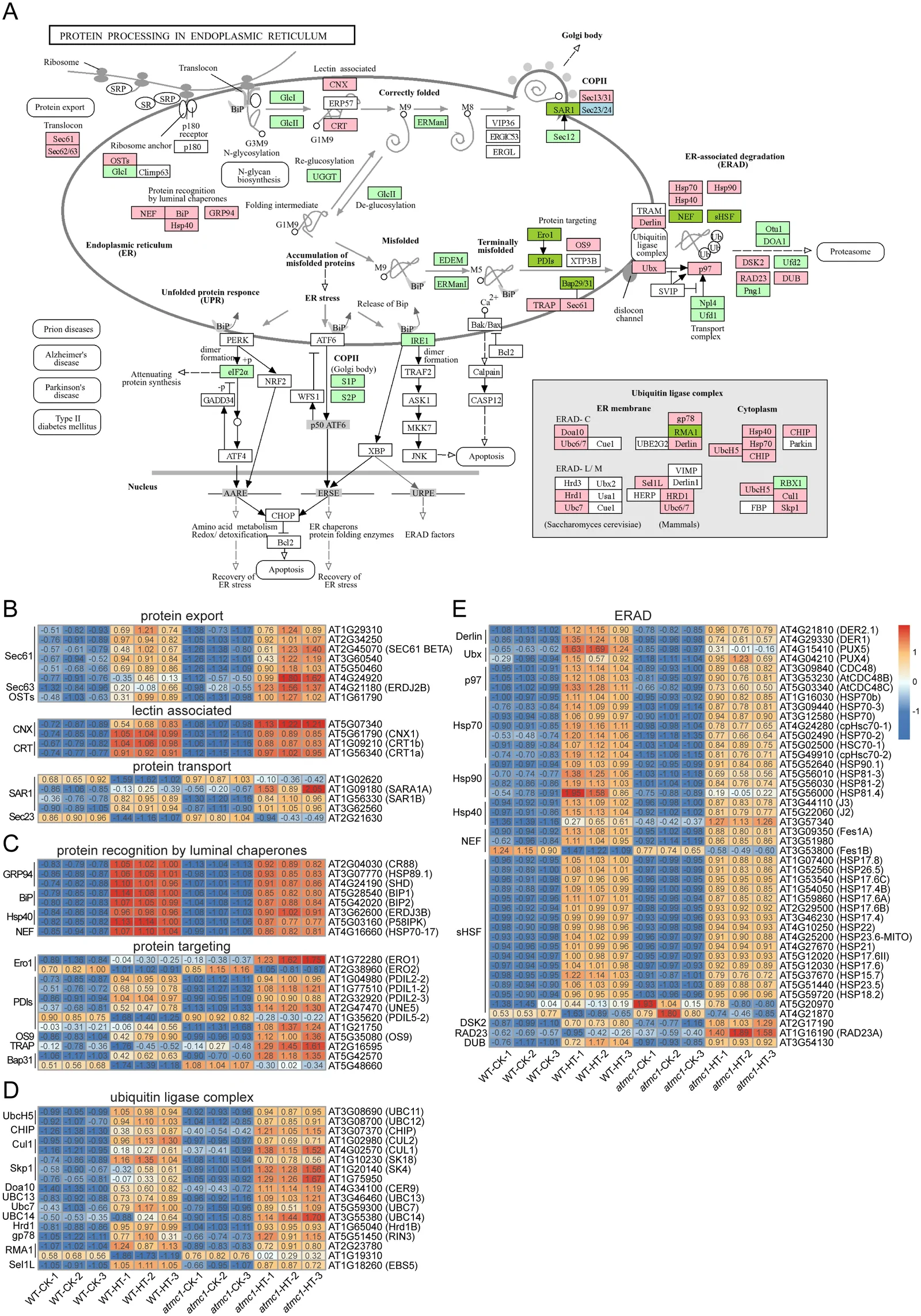
*Protein processing in endoplasmic reticulum* pathway and heatmaps of related genes. **(A)** KEGG pathway map of *protein processing in endoplasmic reticulum.* Pink, light blue, and dark green boxes indicate proteins encoded by DEGs that are upregulated, downregulated, or both, respectively. **(B–E)** Heatmaps showing expression patterns of the DEGs involved in protein folding and degradation. The color scale represents the z-score normalized FPKM values.

In total, 104 genes associated with the pathway *protein processing in endoplasmic reticulum* were identified. These genes were primarily involved in protein translocation, folding, modification, and degradation (**Figure 7A**; **Supplementary Table 11**).

Following heat treatment, the majority of these genes were upregulated (**Figure 7B**). Specifically, all eight genes related to protein translocation, including Sec61, Sec63, and OST components, were heat-induced. Similarly, all four genes encoding calnexin (CNX) and calreticulin (CRT) were upregulated by heat stress. Among the six genes involved in vesicular transport, three (*AT1G09180*, *AT1G56330*, and *AT3G62560*) were upregulated, whereas two (*AT1G02620* and *AT2G21630*) were downregulated. *AT1G18830* was excluded from further analysis because of its low expression level (FPKM < 1).

A total of 21 genes involved in misfolded protein repair, including GRP94, BiP, Hsp40, NEF, Ero1, PDIs, OS9, TRAP, and Bap31, were identified (**Figure 7C**). Most of these genes were induced by heat stress. In contrast, *AT2G38960*/*ERO2*, *AT5G48660* and *AT1G35620*/*PDIL5–*2 were downregulated in both WT and *atmc1*, and *AT2G16595* was specifically upregulated in *atmc1*. Notably, the expression of the glucose-regulated protein 94 (GRP94) gene (*AT3G07770*/*HSP89.1*), BiP genes (*AT5G28540*/*BIP1* and *AT5G42020*/*BIP2*), Hsp40 gene (*AT5G03160*/*P58IPK*) and NEF gene (*AT4G16660*/*HSP70-17*) were moderately reduced in *atmc1*-HT relative to WT-HT. In addition, 19 genes encoding components of the ubiquitin ligase complex, including UbcH5, CHIP, Cul1, Skp1, Doa10, Ubc6/7, Hrd1/HRD1, gp78, RMA1, and Sel1L, were induced by heat stress (**Figure 7D**). An exception was *AT1G19310*, which was downregulated in WT but showed no significant change in *atmc1*.

Terminally, misfolded proteins are retained within the ER lumen through association with molecular chaperones and subsequently targeted for proteasomal degradation via ERAD. We identified 46 genes involved in ERAD, including Derlin, Ubx, p97, Hsp70, HSP90, Hsp40, NEF, sHSF, DSK2, RAD23, and DUB proteins, which function in eliminating misfolded proteins (**Figure 7E**). Except for *AT3G53800*/*Fes1B* and *AT4G21870*, all of these genes were induced by heat treatment. *AT1G56410* was excluded because of its very low expression level (FPKM < 1). Notably, several core ERAD components showed significantly reduced expression in *atmc1* compared to wild type under heat stress, including Derlin (*AT4G29330*/*DER1*), Ubx (*AT4G15410*/*PUX5*), Hsp70 (*AT1G16030*/*HSP70b* and *AT3G09440*/*HSP70-3*), HSP90 (*AT5G52640*/*HSP90.1*, *AT5G56010*/*HSP81-3*, *AT5G56030*/*HSP81-2*), NEF (*AT3G09350*/*Fes1A*), and sHSFs (*AT1G07400*/*HSP17.8*, *AT1G52560*/*HSP26.5*, *AT1G54050*/*HSP17.4B*, *AT1G59860*/*HSP17.6A*, *AT4G25200*/*HSP23.6-MITO*, *AT5G12030*/*HSP17.6*, *AT5G37670*/*HSP15.7*, *AT5G51440*/*HSP23.5).* Additional genes, including *AT3G53230*/*AtCDC48B*, *AT5G02490*/*HSP70-2*, *AT5G56000*/*HSP81.4*, and *AT5G22060*/*J2*, also exhibited slightly reduced expression in *atmc1* under heat treatment.

### 3.8 Expression of heat shock proteins, heat shock transcription factors, and other heat-responsive genes

**Figure 8:**
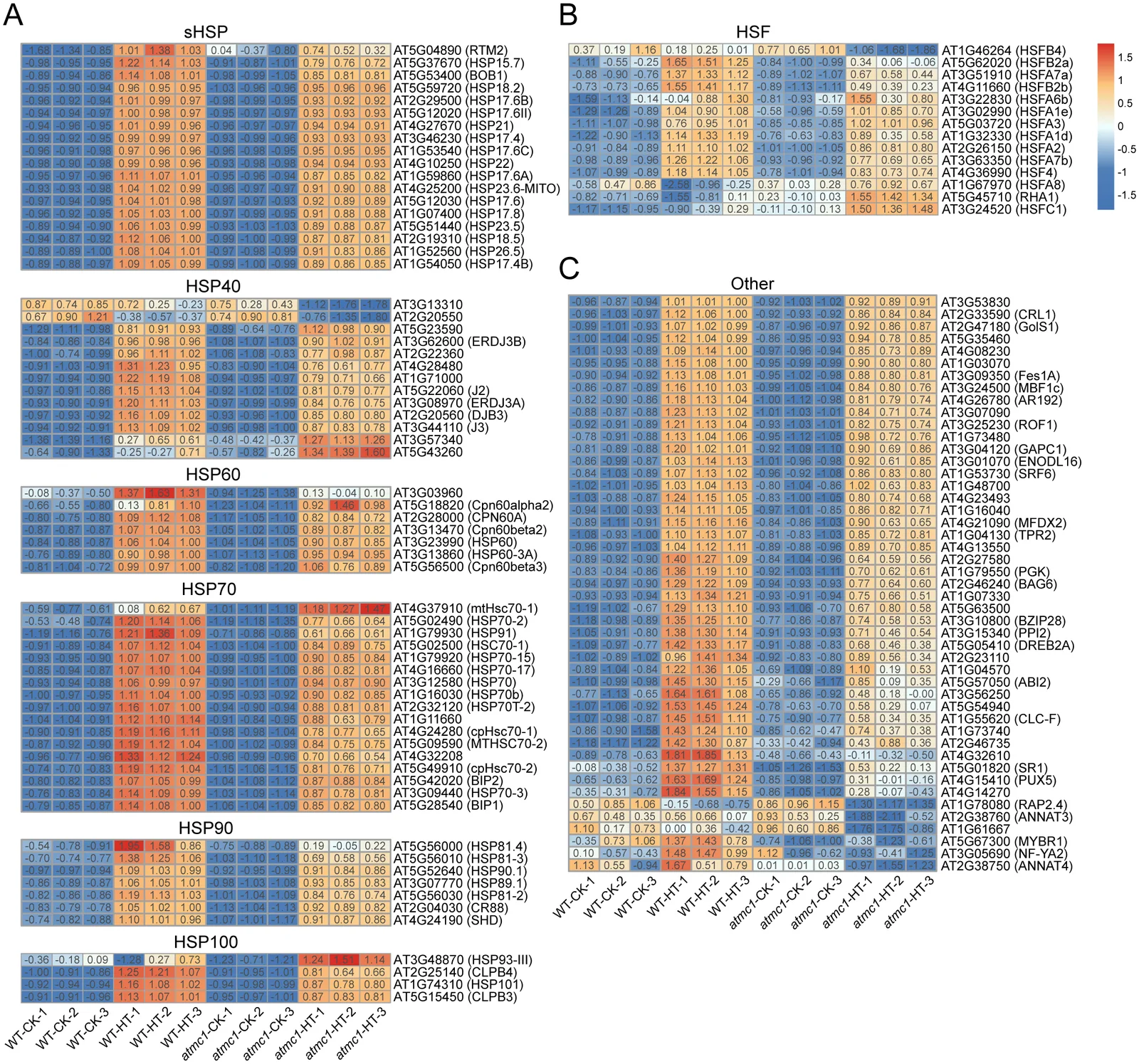
Heatmaps showing expression patterns of selected **(A)** HSP-encoding, **(B)** HSF-encoding, and **(C)** other genes enriched in *response to heat*. The color scale represents the z-score normalized FPKM values.

One of the best-characterized responses to heat stress is the rapid induction of HSP genes, which play essential roles in plant thermotolerance (Queitsch et al., 2000; Sanmiya et al., 2004; Lee et al., 2005; Miroshnichenko et al., 2005; Charng et al., 2006). We identified 66 heat-responsive HSP genes, including 18 sHSPs, 13 HSP40s, 7 HSP60s, 17 HSP70s, 7 HSP90s, and 4 HSP100s, most of which were induced by heat treatment (**Figure 8A**; **Supplementary Table 12**). Several sHSPs, including *HSP15.7*, *HSP17.6A*, *HSP23.6-MITO*, *HSP17.6*, *HSP17.8*, *HSP23.5*, *HSP26.5*, and *HSP17.4B*, as well as HSP40s (*AT1G71000*, *AT3G08970*/*ERDJ3A*, and *AT2G20560*/*DJB3*), HSP70s (*HSP70b*, *AT2G32120*/*HSP70T-2*, *AT5G09590*/*MTHSC70-2*, *AT4G32208*, and *HSP70-3*), HSP90s (*HSP81-3*, *HSP90.1*, and *HSP81-2*), and HSP100s (*AT2G25140*/*CLPB4*, *AT1G74310*/*HSP101*, and *AT5G15450*/*CLPB3*), displayed reduced expression in *atmc1* compared to WT following heat treatment.

The rapid induction of HSP genes under heat stress is primarily mediated by heat shock transcription factors (HSFs). Our analysis identified 14 HSF genes among the heat-responsive DEGs (**Figure 8B**; **Supplementary Table 12**). Among these, *AT1G46264*/*HSFB4* was specifically downregulated by heat stress in *atmc1* but not in WT. Several HSF genes, including *AT1G46264*/*HSFB4*, *AT5G62020*/*HSFB2a*, *AT3G51910*/*HSFA7a*, *AT4G11660*/*HSFB2b*, *AT1G32330*/*HSFA1d*, *AT2G26150*/*HSFA2*, *AT3G63350*/*HSFA7b*, and *AT4G36990*/*HSF4* exhibited significantly lower expression levels in *atmc1* after heat treatment. In contrast, *AT1G67970*/*HSFA8*, *AT5G45710*/*RHA1* and *AT3G24520*/*HSFC1* showed higher expression in *atmc1* than in WT following heat treatment. Furthermore, 47 additional heat-responsive genes exhibited reduced expression in *atmc1* under heat stress conditions (**Figure 8C**; **Supplementary Table 12**). For instance, *AT4G26780*/*AR192* and *AT5G05410*/*DREB2A*, both of which are also involved in *heat acclimation*.

### 3.9 Expression of genes involved in ROS homeostasis

**Figure 9:**
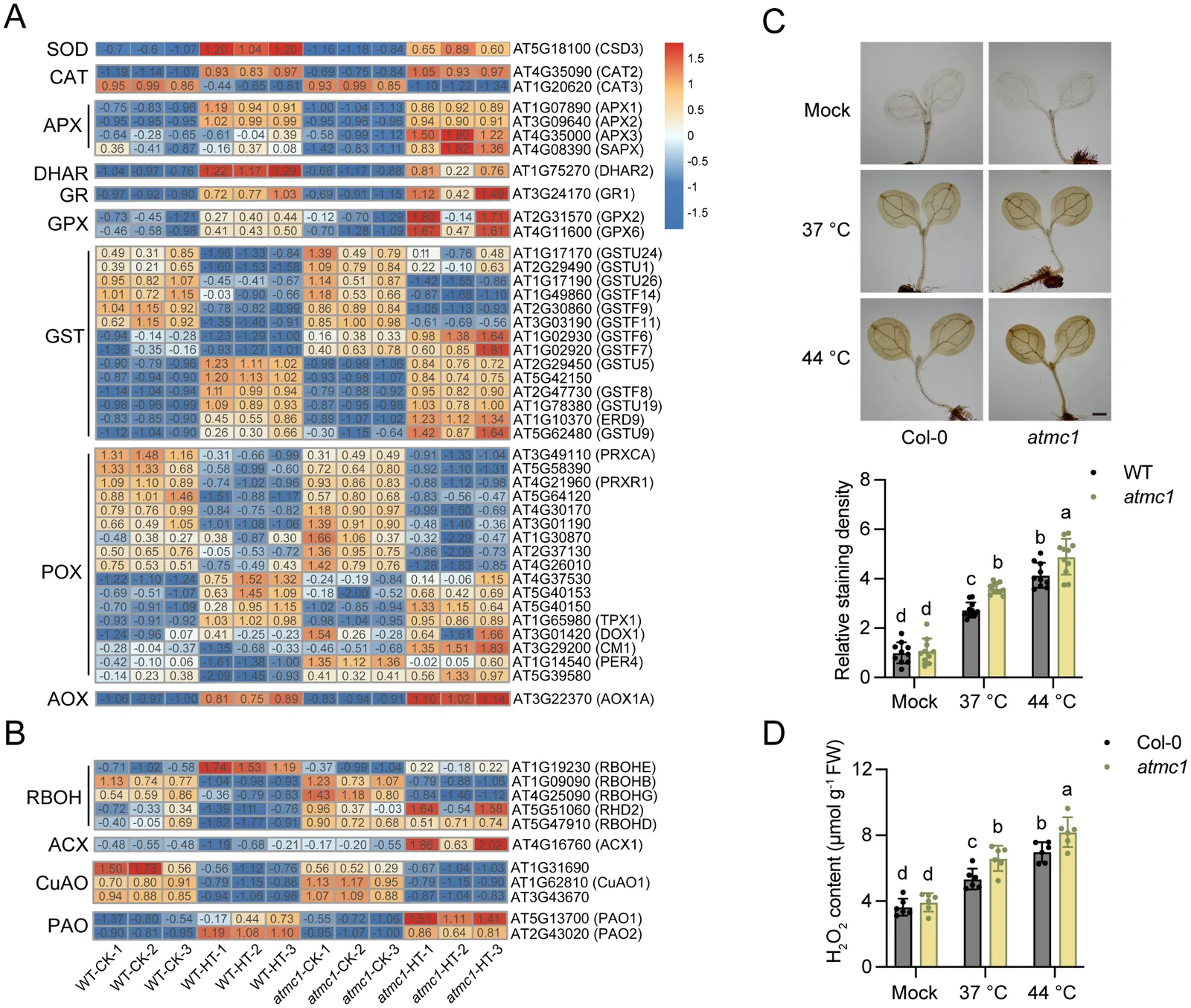
Heat-responsive expression patterns of ROS-related genes and H_2_O_2_ accumulation in WT and *atmc1*. **(A, B)** Heatmaps showing the expression patterns of selected genes involved in **(A)** ROS scavenging and **(B)** ROS production. The color scale represents the z-score normalized FPKM values. POX, peroxidase; AOX, alternative oxidase; CuAO, copper amine oxidase; PAO, polyamine oxidase. **(C)** DAB staining and histochemical analysis of H_2_O_2_ accumulation in cotyledons of 5-day-old seedlings subjected to 37 °C or 44 °C for 1 h. Relative DAB staining intensity was quantified using Fiji, normalized to WT under control conditions. Scale bar = 1 mm. Data are shown as mean ± SD (*n* ≥ 10). **(D)** Quantification of H_2_O_2_ content of whole seedlings. Data are shown as mean ± SD (*n* = 3, approximately 0.1 g fresh weight per sample). Fw, fresh weight. Different letters indicate statistically significant differences determined by two-way ANOVA followed by Tukey’s test (*P* < 0.05).

Under heat stress, ROS function as important signaling molecules that contribute to heat acclimation; however, excessive ROS accumulation can impair thermotolerance. To mitigate heat-induced oxidative stress, plants activate an enzymatic antioxidant network including SOD, CAT, APX, GPX, GR, GST, MDHAR, and DHAR. Under heat stress conditions, core antioxidant enzymes were broadly induced in both WT and *atmc1* (**Figure 9A**; **Supplementary Table 13**). Specifically, *AT5G18100*/*CSD3* and *AT4G35090*/*CAT2* were strongly upregulated by heat, whereas *AT1G20620*/*CAT3* was distinctly downregulated in *atmc1* but remained unchanged in WT (FC = 0.82). Components of the ascorbate-glutathione cycle, including *AT1G07890*/*APX1*, *AT3G09640*/*APX2*, *AT4G35000*/*APX3*, and *AT4G08390*/*SAPX*, *AT1G75270*/*DHAR2*, *AT3G24170*/*GR1*, were induced by heat stress. However, the induction of *APX2* and *DHAR2* was attenuated in *atmc1*, resulting in lower expression levels compared with WT under heat stress. Similarly, *AT2G31570*/*GPX2* and *AT4G11600*/*GPX6* were upregulated in response to heat stress. The GST family exhibited diverse and genotype‑dependent expression patterns. While *AT2G29450*/*GSTU5*, *AT5G42150*, *AT2G47730*/*GSTF8*, *AT1G78380*/*GSTU19*, *AT1G10370*/*ERD9*, and *AT5G62480*/*GSTU9* were heat-inducible in both genotypes, *AT2G30860*/*GSTF9* and *AT3G03190*/*GSTF11* were repressed by heat stress in both genotypes. Notably, *GSTU5* and *AT5G42150* showed attenuated induction in *atmc1*, whereas *ERD9* displayed enhanced induction. In addition, *AT1G02930*/*GSTF6* and *AT1G02920*/*GSTF7* were induced exclusively in *atmc1*.

Peroxidase genes, including *AT3G49110*/*PRXCA*, *AT5G58390*, *AT4G21960*/*PRXR1*, *AT5G64120*, *AT4G30170*, *AT3G01190*, and *AT1G14540*/*PER4* were generally suppressed by heat stress. Among them, the down-regulation of *PER4* was attenuated in *atmc1*. In contrast, several other peroxidase genes, namely *AT4G37530*, *AT5G40153, AT5G40150*, and *AT1G65980/TPX1*, were induced by heat treatment. Notably, *AT3G29200*/*CM1* showed preferential upregulation in *atmc1*. In addition to these classical ROS-scavenging enzymes, the mitochondrial alternative oxidase gene *AT3G22370*/*AOX1A* was also induced by heat stress in both genotypes.

ROS-producing enzymes also exhibited differential expression in response to heat stress (**Figure 9B**; **Supplementary Table 13**). Within the respiratory burst oxidase homolog (RBOH) family, *AT1G19230*/*RBOHE* was induced in both genotypes, although to a lesser extent in *atmc1*; *AT1G09090/RBOHB* and *AT4G25090*/*RBOHG* were repressed in both, with stronger downregulation of *RBOHG* in the mutant; and *AT5G47910*/*RBOHD* was specifically downregulated in WT. Beyond the RBOHs, three copper amine oxidase (CuAO) genes (*AT1G62810*/*CuAO1*, *AT3G43670*, *AT1G31690*) were downregulated by heat stress in both genotypes. Polyamine oxidase genes also displayed differential regulation: induction of *AT2G43020*/*PAO2* was attenuated in *atmc1*, whereas *AT5G13700*/*PAO1* exhibited enhanced heat induction.

To further evaluate ROS-related changes associated with these transcriptomic alterations, we examined H_2_O_2_ accumulation using DAB staining, as H_2_O_2_ is considered a relatively stable ROS species with a comparatively longer half-life. As expected, both WT and *atmc1* seedlings exhibited stronger brown precipitates after heat treatment, indicating increased H_2_O_2_ accumulation. Notably, *atmc1* seedlings accumulated higher levels of H_2_O_2_ than WT, as indicated by darker staining under heat stress conditions (**Figure 9C**). Quantitative analysis revealed no significant difference between Col-0 and *atmc1* under normal growth conditions; however, exposure to 37 °C or 44 °C for 1 h resulted in significantly greater H_2_O_2_ accumulation in *atmc1* compared with Col-0 (**Figure 9D**). Collectively, these observations suggest that loss of *AtMC1* is associated with increased H_2_O_2_ accumulation under heat stress conditions.

### 3.10 Identification of core transcription factors in response to heat stimulus

**Figure 10:**
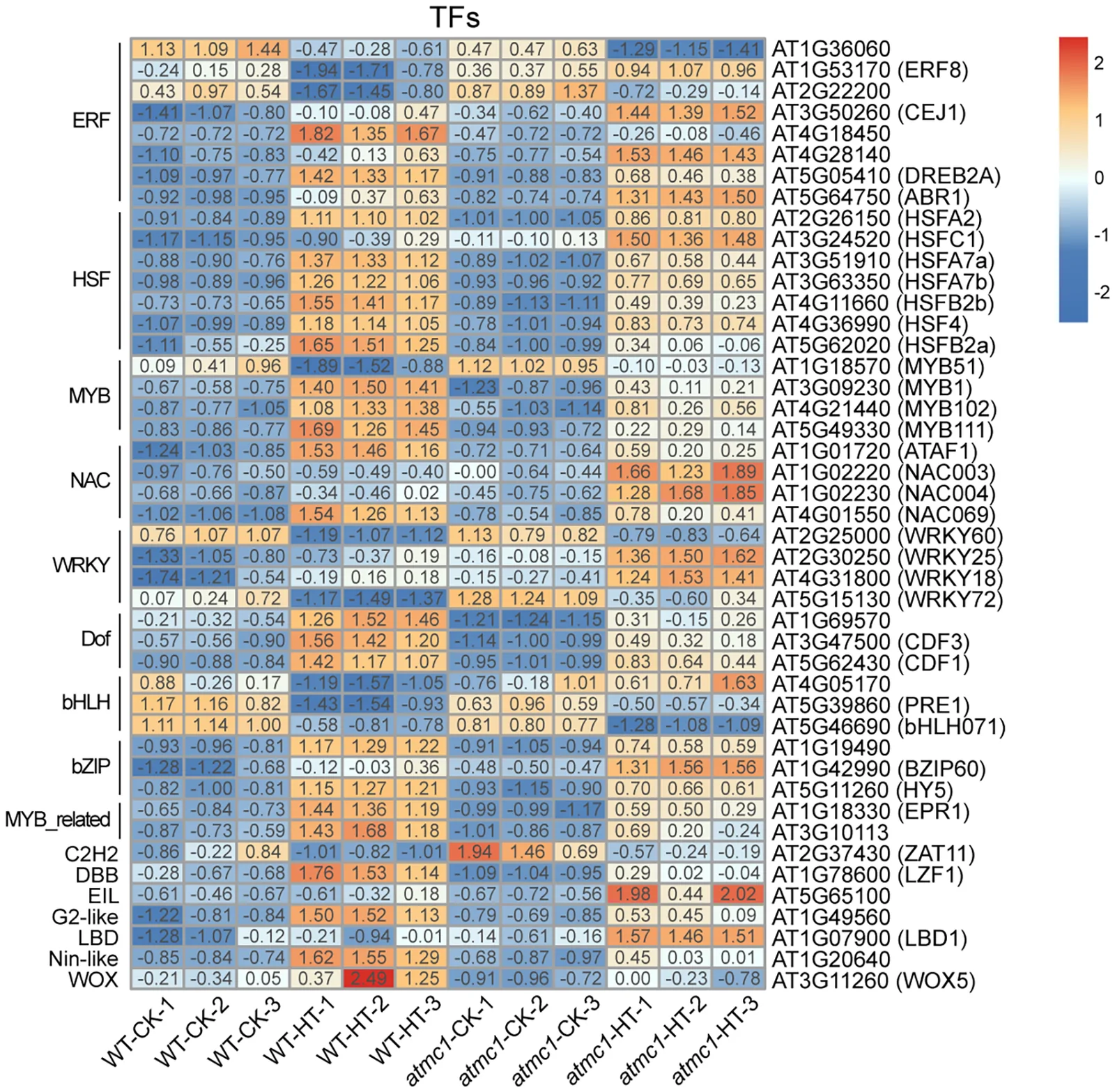
Heatmap of differentially expressed transcription factors. Expression patterns of gene members from the ERF, HSF, MYB, NAC, WRKY, Dof, bZIP, and other TF families. The color scale represents the z-score normalized FPKM values.

From 6,254 DEGs identified in WT under heat stress (WT-HT vs. WT-CK), 467 genes encoding TFs were detected, belonging to 46 TF families. The most abundant TF families (each > 10 members) were ERF (46), bHLH (39), MYB (35), NAC (34), C2H2 (33), bZIP (30), MYB-related (26), WRKY (19), B3 (17), HD-ZIP (17), C3H (13), HSF (12), and Dof (11). In the *atmc1* mutant, 421 genes encoding TFs were identified from 5,561 DEGs, with a similar family distribution (**Supplementary Table 14**). Comparison of the two TF sets revealed 284 common TF genes, among which the top 10 families were ERF, MYB, NAC, bHLH, C2H2, MYB-related, B3, bZIP, HSF and WRKY. To identify TFs potentially responsible for the altered heat responses in *atmc1*, the 284 common TFs were intersected with the 177 TFs differentially expressed in *atmc1*-HT vs. WT-HT, yielding 45 overlapping TF genes for further analysis. Interestingly, most of these 45 TF genes exhibited lower expression levels in *atmc1* than in WT following heat stress (**Figure 10**; **Supplementary Table 14**).

### 3.11 Prediction of PPI networks to identify the hub genes in response to heat treatment

**Figure 11:**
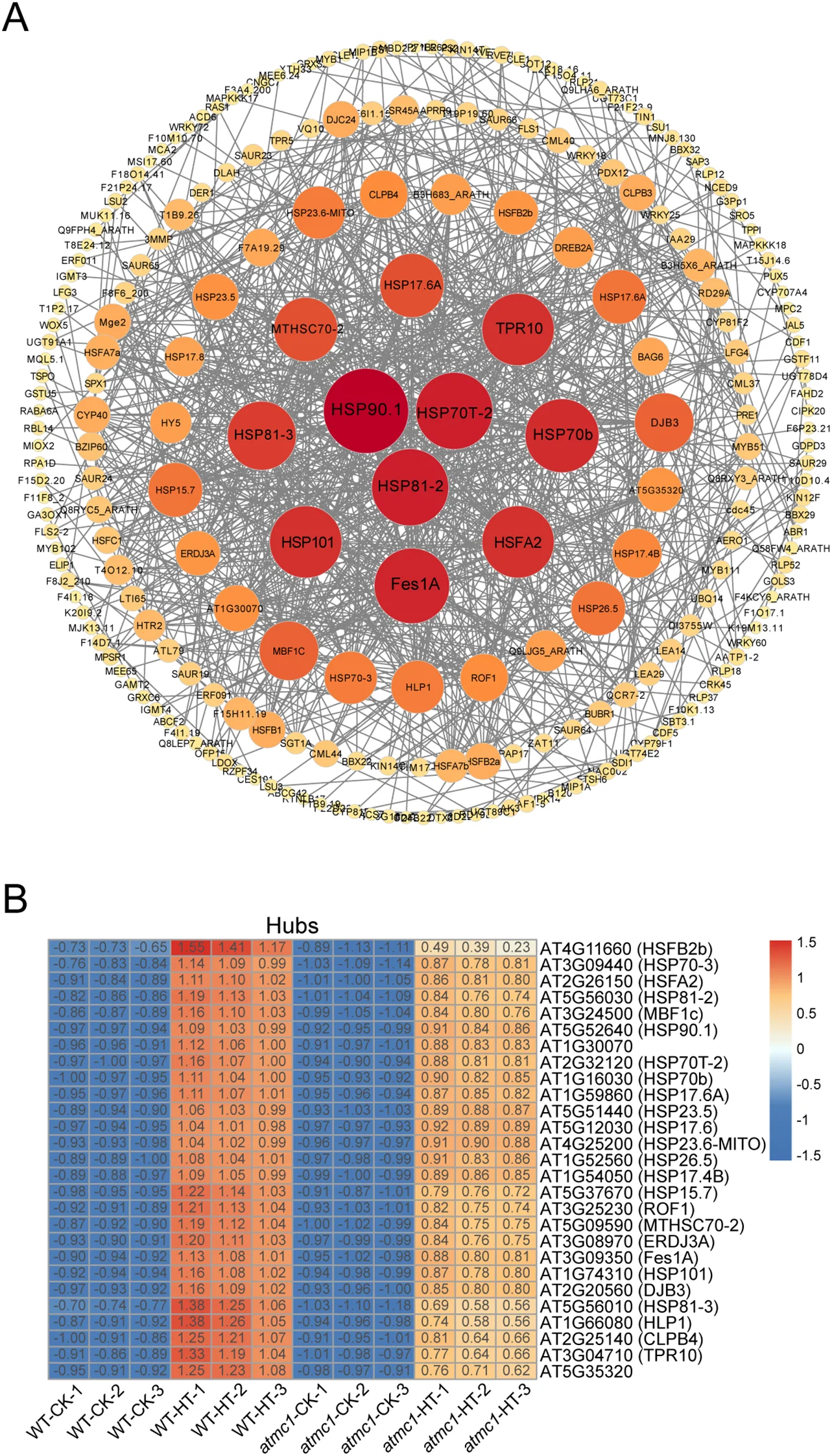
PPI analysis of the 479 common DEGs between WT and *atmc1*. **(A)** Protein–protein interaction network constructed using the STRING database. **(B)** Expression patterns of the 27 hub genes (Hubs). The color scale represents the z-score normalized FPKM values.

To identify key genes associated with heat stress responses, we focused on 479 genes that were differentially expressed in the three pairwise comparisons: WT-HT vs. WT-CK, *atmc1*-HT vs. *atmc1*-CK, and *atmc1*-HT vs. WT-HT (**Figure 3C**). PPI network analysis using the STRING database (**Supplementary Data Sheet 3**) identified 27 hub genes among these 479 genes (**Figure 11A**; **Supplementary Table 15**).

The expression patterns of these hub genes are shown in the heatmap (**Figure 11B**). All 27 hub genes were upregulated in both genotypes under heat stress conditions. These include 18 HSP genes, 2 HSF genes, and 3 co-chaperone genes (*Fes1A*, *AT3G04710*/*TPR10*, *AT3G25230*/*ROF1*). Additional hub genes included *AT3G24500*/*MBF1c*, a transcriptional co-activator involved in heat tolerance through partial activation of the ethylene-response signaling pathway (Tsuda et al., 2004; Suzuki et al., 2005, Suzuki et al., 2008); *AT1G66080*/*HLP1*, a glucose-regulated protein associated with thermotolerance and thermomemory (Koizumi et al., 2014; Sharma et al., 2019); *AT5G35320*, which encodes a DBH-like monooxygenase; and *AT1G30070*, an SGS domain-containing protein. Collectively, these hub genes are likely to play pivotal roles in the transcriptional and regulatory networks activated by heat stress. Notably, all of them exhibited lower expression in *atmc1* than in WT after heat treatment.

### 3.12 Expression pattern of *AtMC1* and validation of RNA-seq results by RT-qPCR

**Figure 12:**
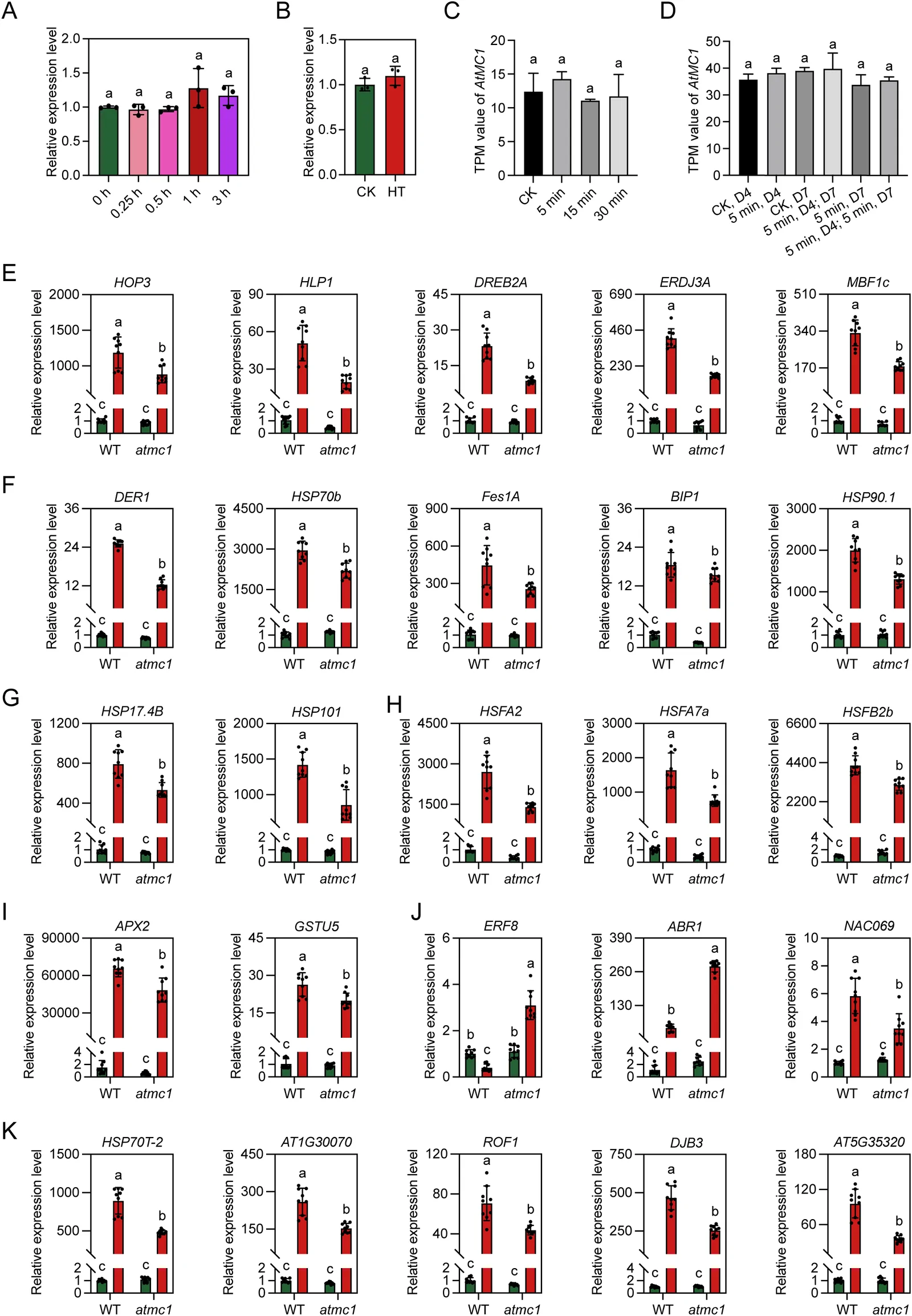
Expression patterns of *AtMC1* and RT-qPCR validation of selected genes identified by RNA-seq analysis. Analysis of *AtMC1* expression using **(A)** 7-day-old seedlings under different durations of 37 °C heat treatment and **(B)** RNA-seq samples. Data are presented as mean ± SD from technical replicates. **(C, D)** Expression profiles of *AtMC1* under heat stress based on the public dataset GSE260655 (Peng et al., 2025), which provides a shorter and higher-resolution heat treatment time course. D4/7 indicates day 4 or day 7. Data are presented as mean ± SD from biological replicates. Statistical significance was determined by one-way ANOVA **(A, C, D)** or Welch’s *t*-test **(B)**; the same letters indicate no significant differences (*P* ≥ 0.05). Genes associated with **(E)** response to heat and **(F)** protein processing in endoplasmic reticulum, as well as **(G)** HSP-encoding genes, **(H)** HSF-encoding genes, **(I)** ROS-scavenging genes, **(J)** TF-encoding genes, and **(K)** hub genes. Data are shown as mean ± SD from three biological replicates, each with three technical replicates. Different letters indicate significant differences determined by two-way ANOVA followed by Tukey’s test (*P* < 0.05).

The above results have shown that *AtMC1* is involved in thermotolerance regulation. To determine whether *AtMC1* is heat-inducible, we examined its expression pattern under heat stress. As shown in **Figure 12A**, treatment at 37 °C for 3 h did not induce *AtMC1* expression. RNA-seq validation of *AtMC1* expression in the RNA-seq samples (**Figure 12B**), together with the time-course heat treatment experiments spanning 24 h from Publicly available eFP (https://bar.utoronto.ca/efp//cgi-bin/efpWeb.cgi) and GEO datasets (https://ncbi.nlm.nih.gov/geo/query/acc.cgi?acc=GSE260655) (**Figures 12C, D**), consistently supported that *AtMC1* expression remained largely stable during heat treatment. To further corroborate the RNA-seq results, we also examined the expression of representative heat-responsive genes using RT-qPCR. These included genes involved in *response to heat* and *heat acclimation* (**Figure 12E**); *protein processing in the endoplasmic reticulum* (**Figure 12F**); HSP-encoding genes (**Figure 12G**); HSF-encoding genes (**Figure 12H**); ROS-scavenging genes (**Figure 12I**); transcription factors (**Figure 12J**); and hub genes (**Figure 12K**). The results confirmed that nearly all of these genes were strongly induced by heat treatment but their induction was attenuated in the *atmc1* relative to WT.

## 4 Discussion

Metacaspases are a conserved family of cysteine proteases in plants implicated in regulating programmed cell death and senescence (Coll et al., 2010; Tsiatsiani et al., 2011; Coll et al., 2014; Sueldo and van der Hoorn, 2017; Klemencic and Funk, 2019; Basak and Kundu, 2022; Salas-Gómez et al., 2026). However, their roles and regulatory mechanisms in abiotic stress remain largely unexplored, as previous studies have focused almost exclusively on biotic stress. Notably, Ruiz-Solani et al. reported that the *atmc1* knockout mutant did not exhibit altered thermotolerance compared with WT, although they had predicted such a phenotype (Ruiz-Solani et al., 2023). In contrast, our study, using the same T-DNA insertion line, revealed a clear heat-sensitive phenotype in *atmc1* seedlings. Basal thermotolerance assays showed a substantially reduced survival rate of *atmc1* seedlings under heat stress, whereas acquired thermotolerance was only moderately affected under our experimental conditions (**Figure 1**). Genetic complementation further confirmed the role of AtMC1 in thermotolerance (**Figure 2**). Together, these results suggest that AtMC1 plays a more prominent role in basal thermotolerance than in heat acclimation-mediated acquired thermotolerance. The discrepancy between our study and those of Ruiz-Solani et al. may be attributed to differences in experimental conditions, such as seedling developmental stage and heat stress treatment regimes. Interestingly, despite not observing the heat-sensitive phenotype in the *atmc1* mutant, Ruiz-Solani et al. found that *AtMC1* overexpression conferred enhanced thermotolerance (Ruiz-Solani, 2025), further supporting its positive role under heat stress.

To dissect the global molecular alterations associated with AtMC1 under heat stress, we performed RNA-seq analysis. Heat treatment triggered substantial transcriptional changes in both WT and *atmc1* seedlings. GO analysis revealed pronounced basal transcriptional differences between *atmc1* and WT under control conditions. DEGs in *atmc1*-CK vs. WT-CK were predominantly enriched in defense- and immunity-related processes (**Supplementary Figure 3**), consistent with the known role of AtMC1 in plant immunity (Wang et al., 2021). Notably, the upregulated DEGs were enriched in *leaf senescence* (GO:0010150), consistent with the early senescence phenotype of *atmc1* (Coll et al., 2014). Moreover, even without heat stress, heat-responsive genes were downregulated in *atmc1* (**Supplementary Figure 3**), suggesting compromised basal heat-response capacity. KEGG analysis further showed that heat stress in WT primarily activated pathways associated with protein homeostasis and transcriptional regulation while suppressing growth and secondary metabolism. In *atmc1*, however, heat stress additionally induced phenylpropanoid biosynthesis, protein export, MAPK signaling, and plant–pathogen interaction, accompanied by broader suppression of secondary metabolism (**Figure 5**). GSEA further demonstrated that heat-responsive gene sets, including *heat acclimation*, *protein folding*, and *cellular response to heat*, were downregulated in *atmc1* (**Figure 6**). Collectively, these results suggest that loss of *AtMC1* perturbs transcriptional programs governing heat acclimation and proteostasis.

Protein misfolding and aggregation are markedly exacerbated under heat stress, particularly in the ER. Analysis of the ER protein processing pathway identified 104 DEGs involved in translocation, folding, modification, and degradation (**Figure 7**; **Supplementary Figure 3**). In WT, most of these genes were strongly upregulated upon heat stress, whereas *atmc1* showed attenuated induction of several key ERAD components, including *HSP70b*, *HSP90.1*, *HSP81-2/3*, and the co-chaperones *Fes1B and TRAP*. Although some ERAD-related genes, like *ERO1* and *Bap31*, were upregulated in *atmc1*, these changes appeared insufficient to restore proteostasis, suggesting that impaired chaperone and ERAD regulation contribute to the heat-sensitive phenotype of the *atmc1* mutant.

HSPs and HSFs are central regulators of protein homeostasis and thermotolerance. In WT, heat stress strongly induced multiple HSP families, including sHSPs, HSP40s, HSP60s, HSP70s, HSP90s, and HSP100s (**Figure 8A**). In contrast, *atmc1* showed attenuated induction of several key HSPs, including *HSP17.6A*, *HSP23.6-MITO*, *HSP70b*, *HSP90.1*, and *HSP101*, as well as HSFs such as *HSFA2*, *HSFA7a*/*b*, and *HSFB2a*/*b* (**Figure 8B**).

Under heat stress, ROS levels increase dramatically (Mittler et al., 2022). Plants maintain a dynamic balance between ROS generation and scavenging to enable signaling while minimizing oxidative damage (Volkov et al., 2006; Waszczak et al., 2018). Genes involved in ROS homeostasis responded strongly to heat (**Figures 9A, B**; **Supplementary Table 12**). Although both WT and *atmc1* activated ROS-scavenging pathways, attenuated induction of antioxidant genes such as *APX2*, *DHAR2*, and *GSTU5* in *atmc1* suggests reduced ROS detoxification capacity. Meanwhile, altered regulation of ROS-producing enzymes, including *RBOHD* and *PAO1*, may further influence ROS dynamics in the mutant. Consistently, DAB staining revealed increased H_2_O_2_ accumulation under heat stress (**Figure 9C**), with significantly higher levels in *atmc1* (**Figure 9D**), likely contributing to its heat sensitive phenotype. Nevertheless, as H_2_O_2_ represents only one type of ROS, the present data provide only preliminary evidence of altered ROS homeostasis.

Although *AtMC1* is involved in thermotolerance, its expression does not appear to be induced by heat (**Figure 12**). Similarly, Ruiz-Solani et al. reported heat-induced relocalization of the AtMC1 protein to stress granules rather than transcriptional upregulation (Ruiz-Solani et al., 2023), suggesting that *AtMC1* does not function as a classical heat-inducible gene. Instead, its contribution to thermotolerance may involve mechanisms beyond transcriptional induction, such as changes in protein activity, subcellular localization, or post-transcriptional and post-translational regulation. Although AtMC1 is not a canonical transcriptional regulator, it may indirectly influence transcriptional reprogramming through changes in cellular proteostasis or proteolytic processing of stress-associated signaling components. Consistent with this possibility, excessive H_2_O_2_ accumulation and impaired induction of molecular chaperones in *atmc1* seedlings may further contribute to the transcriptomic alterations observed in this study. Given that metacaspases are proteolytic enzymes, future investigation into AtMC1 protein dynamics, enzymatic activation, substrate specificity, post-translational modifications and signaling partners will be essential for elucidating the molecular basis of its role in thermotolerance. Moreover, the present study focused primarily on the seedling stage, whereas publicly available eFP expression data indicate that *AtMC1* is broadly expressed across tissues and developmental stages, with relatively high expression in floral tissues during reproduction (Schmid et al., 2005; Klepikova et al., 2016). This expression pattern raises the possibility that AtMC1 may also contribute to heat responses beyond seedling thermotolerance, particularly during flowering, which warrants further investigation.

## 5 Conclusion

**Figure 13:**
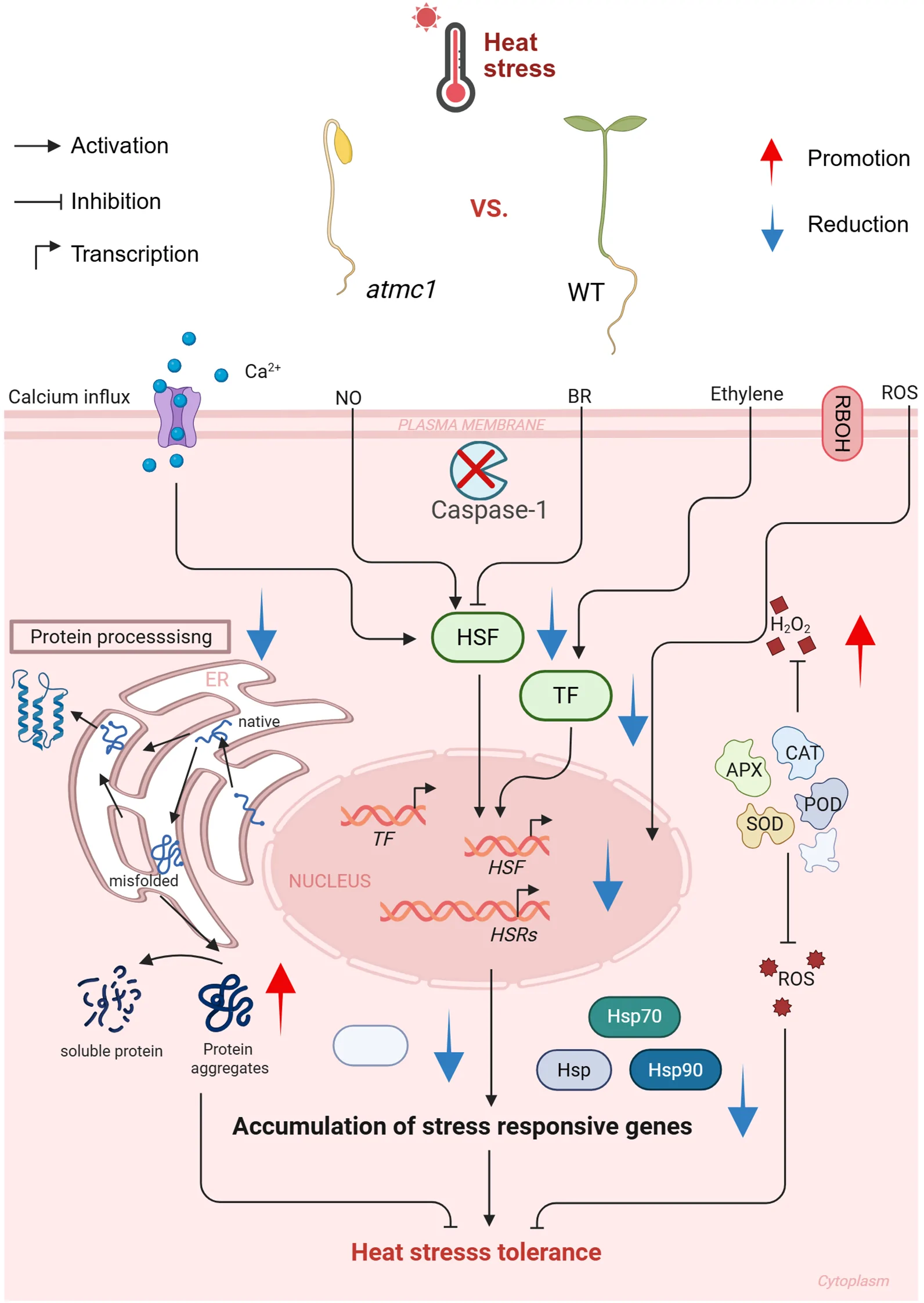
Proposed model of *AtMC1*-associated heat stress responses in *Arabidopsis* seedlings inferred from transcriptomic analysis.

Our study shows that loss of *AtMC1* compromises basal thermotolerance in *Arabidopsis* seedlings, associated with increased H_2_O_2_ accumulation and attenuated induction of genes involved in heat stress responses, ER protein processing, ROS scavenging, and transcriptional regulation. Complementation assays confirmed that restoring *AtMC1* expression rescues the heat-sensitive phenotype. We therefore propose a model in which AtMC1 contributes to heat stress tolerance through these interconnected processes (**Figure 13**). While the precise molecular mechanisms linking AtMC1 activity to these responses remain unclear, our findings extend the functional scope of plant metacaspases to heat tolerance, beyond their well-established roles in immunity and senescence.

## Data Availability

The RNA-seq data are publicly available in the NCBI SRAdatabase under accession number PRJNA1473612.
