# Supplementary material for: Transcriptomic analysis of reduced thermotolerance in Arabidopsis seedlings lacking the metacaspase AtMC1
Source: Front Plant Sci. 2026 Sep 9;17:1873366. doi: 10.3389/fpls.2026.1873366 (PMC13597399; doi:10.3389/fpls.2026.1873366)
Supplement: Supplementary file 1 [file Presentation1.pdf]

Supplementary Material

1 Supplementary Data

Supplementary Data 1. DEGs

Supplementary Data 2. Functional enrichment

Supplementary Data 3. PPI

2 Supplementary Figures and Tables

2.1 Supplementary Figures

A

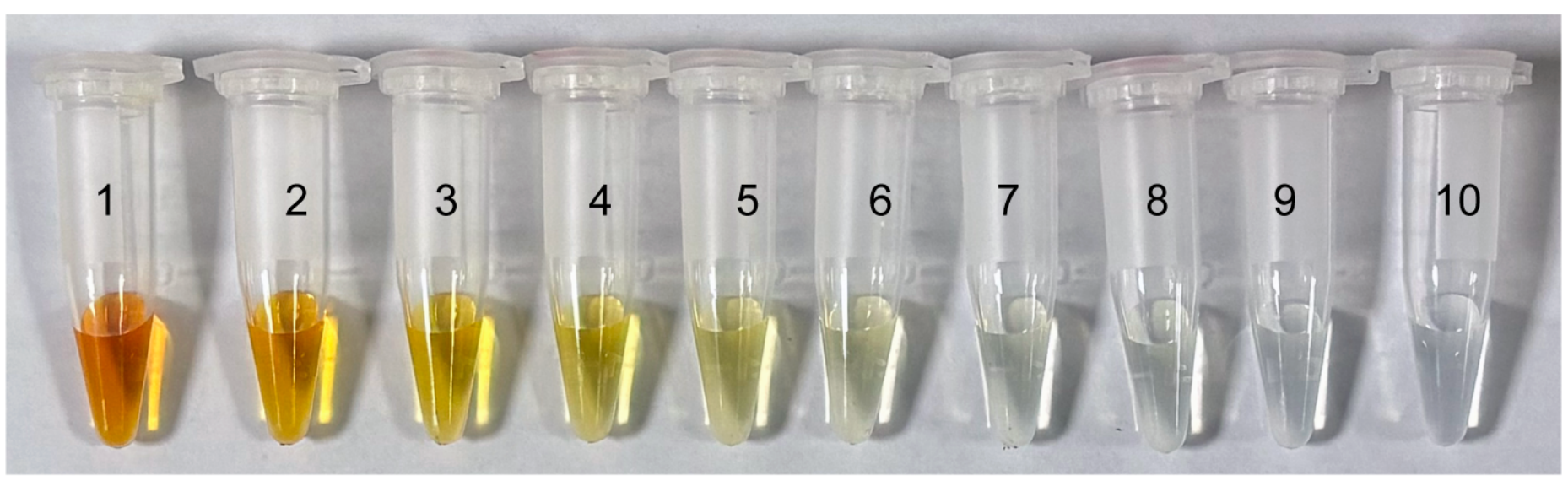

B

ID	H_2O_2 (mM)	A415
1	20	5.9491
2	10	3.6207
3	5	1.9115
4	2.5	0.9701
5	1.25	0.4599
6	0.625	0.2984
7	0.3125	0.1475
8	0.15625	0.0767
9	0.078125	0.0367
10	0.0390625	0.0163

C

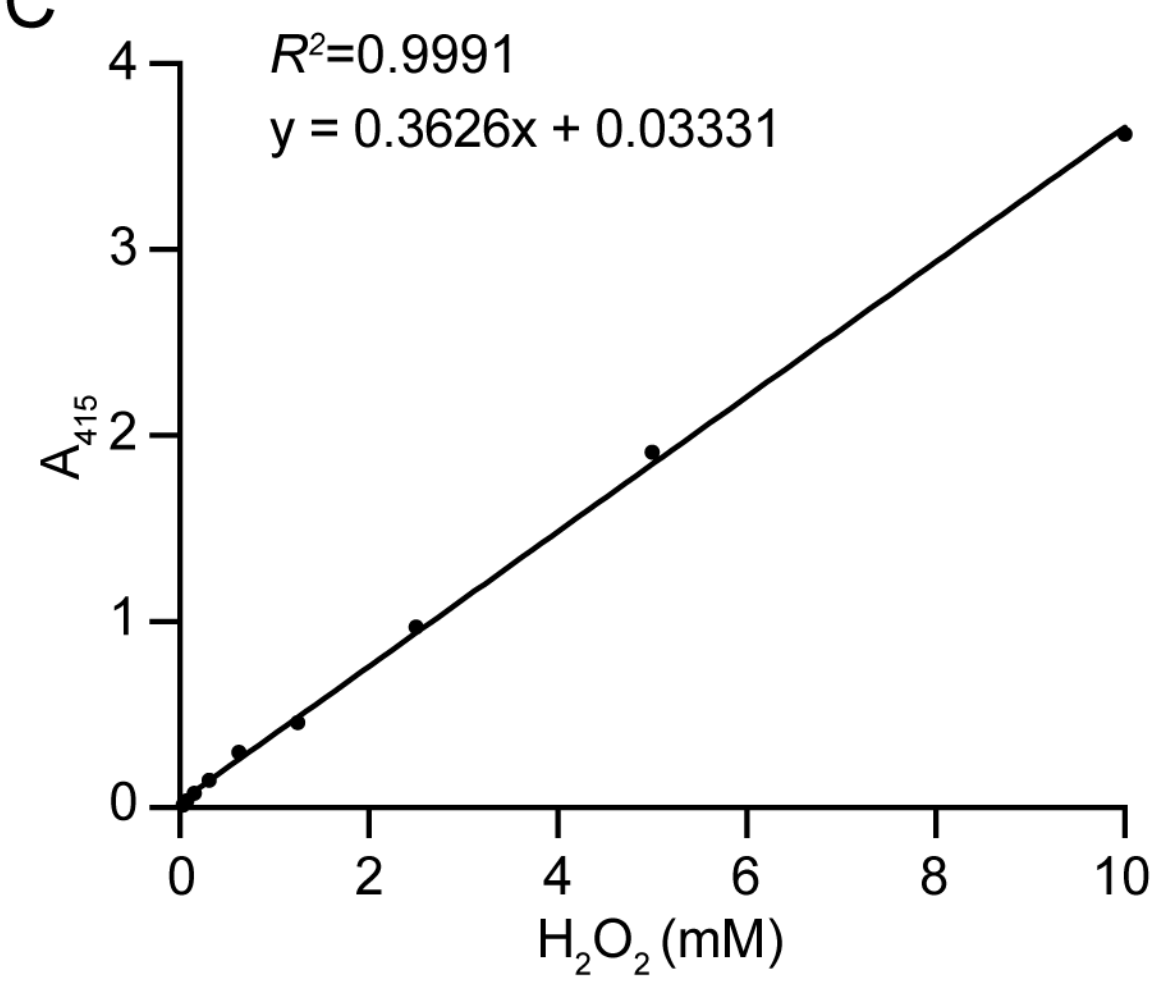

Supplementary Figure 1. Standard curve for the determination of H_2O_2 content using the titanium sulfate colorimetric method. (A) Colorimetric reaction mixtures prepared from serially diluted H_2O_2 standards. (B) Corresponding absorbance values at 415 nm (A_{415}) of the reaction mixtures shown in (A). (C) Standard curve showing the relationship between H_2O_2 concentration and A_{415}.

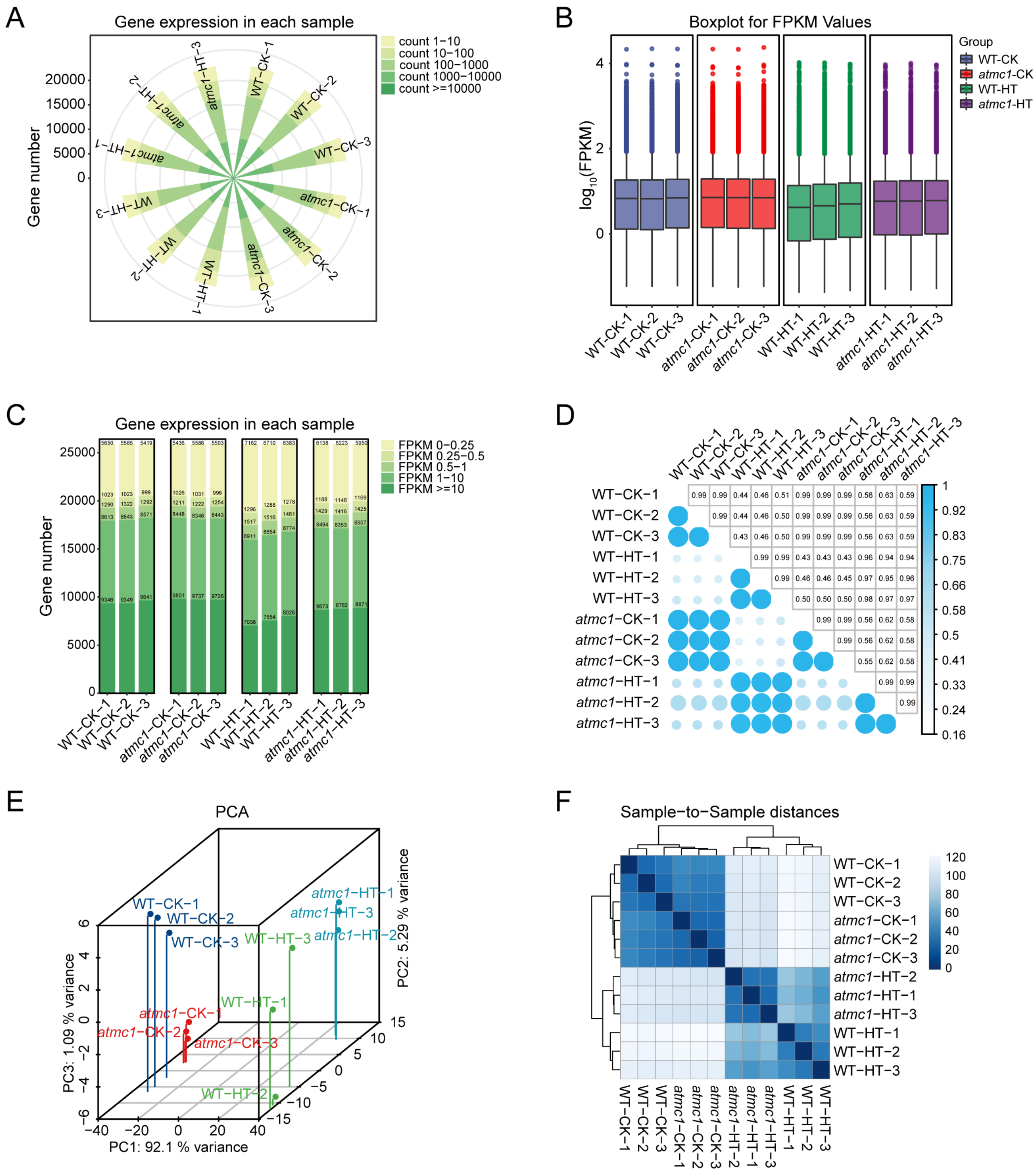

Supplementary Figure 2. Quality assessment and reproducibility of RNA-seq data. (A) Detected gene numbers, (B) box-and-whisker plots of log_{10}(FPKM) values, (C) FPKM distribution across samples, (D) Pearson's correlation coefficient (PCC) analysis, (E) Principal component analysis (PCA), and (F) Hierarchical cluster analysis (HCA) plots.

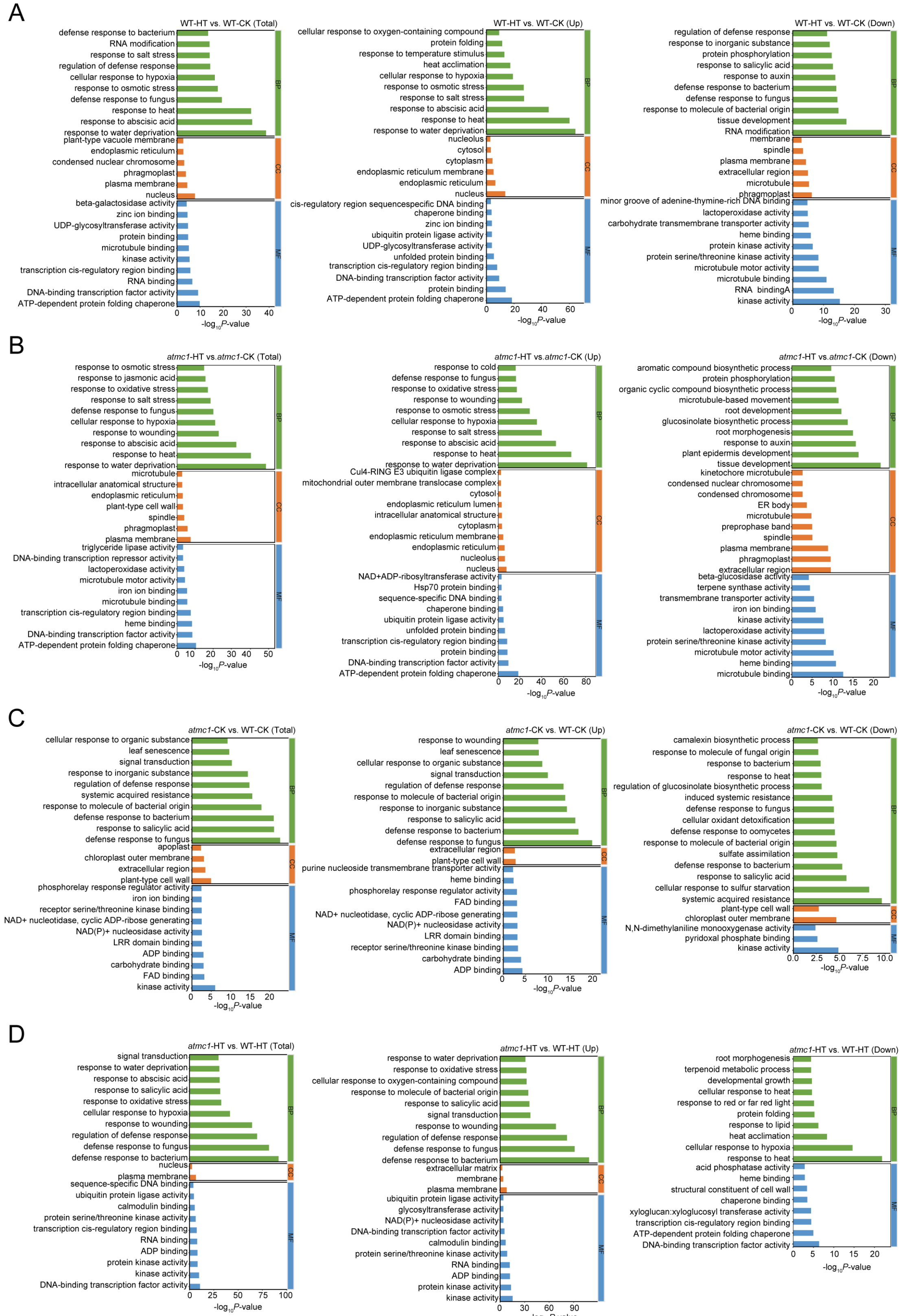

Supplementary Figure 3. Top GO terms for DEGs in four pairwise comparisons: (A) WT-HT vs. WT-CK, (B) *atmc1*-HT vs. *atmc1*-CK, (C) *atmc1*-CK vs. WT-CK, and (D) *atmc1*-HT vs. WT-HT. BP, biological process; MF, molecular function; CC, cellular component.

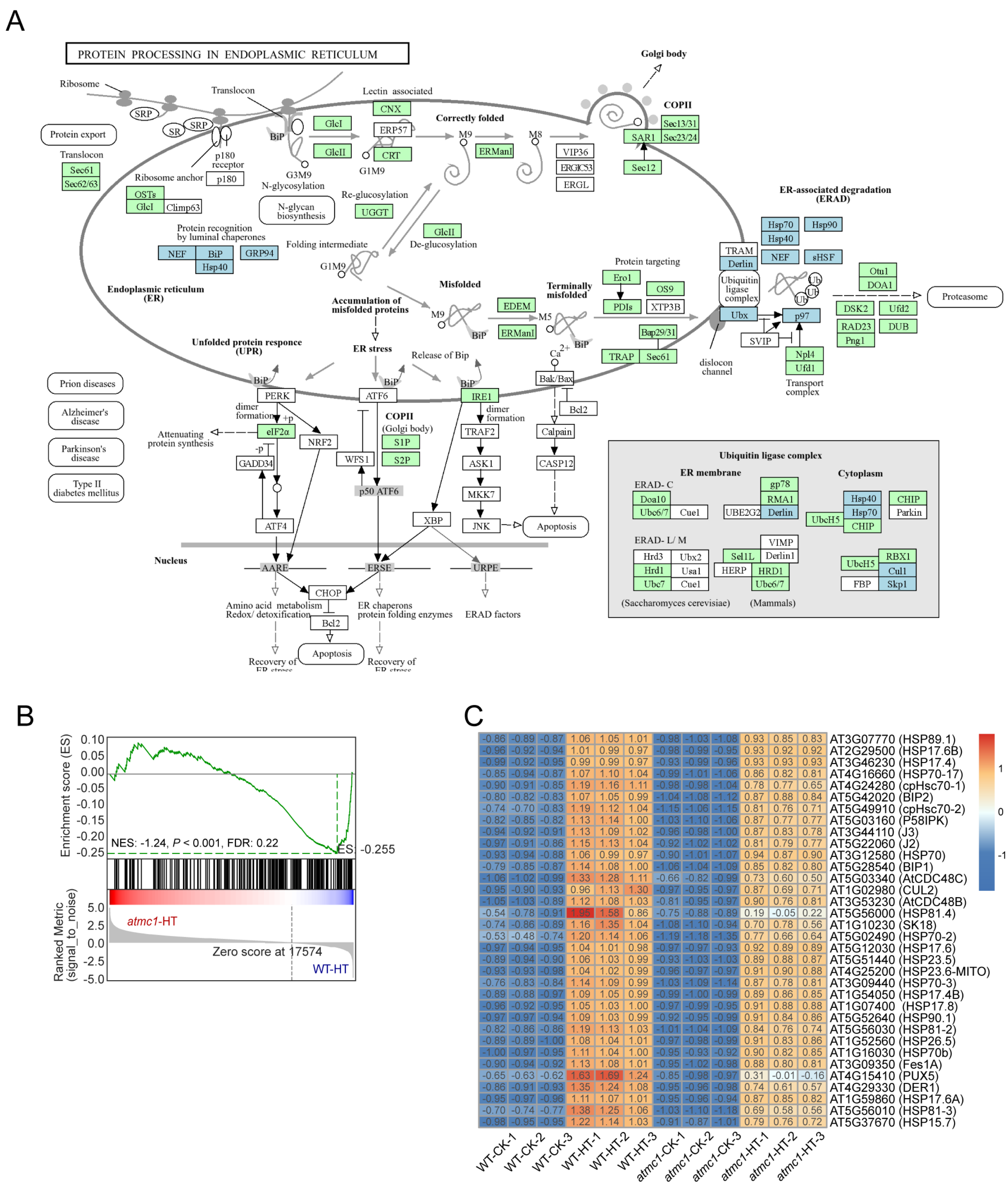

Supplementary Figure 4. *Protein processing in endoplasmic reticulum* pathway and heatmap of related genes. (A) KEGG pathway map of the *protein processing in endoplasmic reticulum*. Proteins highlighted in light blue correspond to genes with lower expression in *atmc1*-HT than in WT-HT. (B) GSEA of protein *processing in endoplasmic reticulum*. (C) Heatmap showing the expression patterns of the 34 core DEGs. The color scale represents the z-score normalized FPKM values.

2.2 Supplementary Tables

Supplementary Table 1. Primers used in this study;

Supplementary Table 2. Summary of RNA-seq data quality and mapping statistics；

Supplementary Table 3. Number of expressed genes detected in each RNA-seq sample；

Supplementary Table 4. Summary of significant DEGs across pairwise comparisons；

Supplementary Table 5. Six gene expression clusters of heat-induced genes (fold change > 2, $q < 0.05$), related to Figure 3；

Supplementary Table 6. Summary of GO term enrichment across pairwise comparisons ($q < 0.05$)；

Supplementary Table 7. GO enrichment analysis of biological process terms for each gene cluster (C1–C6) ($q < 0.05$)；

Supplementary Table 8. Summary of KEGG pathway enrichment across pairwise comparisons ($q < 0.05$)；

Supplementary Table 9. KEGG enrichment analysis for each gene cluster (C1–C6) ($q < 0.05$)；

Supplementary Table 10. Identification of GSEA core genes across pairwise comparisons ($q < 0.05$)；

Supplementary Table 11. Identification of DEGs enriched in *protein processing in endoplasmic reticulum* ($q < 0.05$);

Supplementary Table 12. Identification of DEGs encoding HSPs, HSFs, and other genes enriched in *response to heat*；

Supplementary Table 13. Identification of DEGs encoding enzymes involved in ROS homeostasis；

Supplementary Table 14. Identification of DEGs encoding TFs；

Supplementary Table 15. Identification of hub genes.
